# Understanding the demographics of the opioid overdose death crisis

**DOI:** 10.1007/s00148-025-01108-0

**Published:** 2025-06-19

**Authors:** David Powell

**Affiliations:** RAND, Arlington, VA USA

**Keywords:** Overdose deaths, Racial disparities, OxyContin, Purdue pharma, Deaths of despair, I12, I18, J11, J15

## Abstract

The United States is facing an unprecedented drug overdose crisis, distinguished from prior epidemics by its severity, widespread impact, and demographic incidence. This study examines demographic overdose trends during the opioid crisis relative to historical disparities. Using a simple decomposition framework, I assess the contributions of specific demographic factors while accounting for other characteristics, finding that many disparities intensify when adjusted for covarying factors. Additionally, I analyze the long-term impacts of OxyContin’s launch, discovering that it induced substantial differences in overdose rates by sex and education. While OxyContin contributed to some racial/ethnic disparities, there is evidence that other factors independently shaped these trends. Nonetheless, all demographic groups were substantially impacted by the introduction and promotion of OxyContin. This research highlights how certain groups have been disproportionately affected by the opioid crisis and the foundational role of supply-side shocks to opioid access in explaining the demographics of the overdose death crisis.

**Supplementary Information:**

The online version contains supplementary material available at 10.1007/s00148-025-01108-0.

## Introduction

Drug overdose deaths topped 107,000 in the United States in 2023, over 75% of which involved opioids (Centers for Disease Control and Prevention 2024). The opioid crisis is a national emergency, marked in contrast to previous drug epidemics by its severity, its breadth, and its demographics. All demographic groups have experienced increases in overdose death rates; however, the epidemic has flipped some pre-existing disparities in overdose death rates while exacerbating others. Our understanding of this differential incidence is limited, and little work has explored underlying determinants of this variation, despite the importance of examining such disparities in the context of the opioid crisis (Om 2018). While the literature has provided hypotheses about why certain demographic groups have been more impacted than others, there have been few empirical tests and analyses providing further understanding of these differences. This paper begins to address these issues and to discuss possible driving factors.

The literature has documented that growth in overdose rates has differed substantially by race and ethnicity (Alexander et al. 2018; Tipps et al. 2018), sex (Harris 2022), and education (Xu et al. 2024; Powell 2023).^1^ However, it is unknown to what extent these unadjusted differences reflect disparities due to other demographic factors given correlations between race/ethnicity, the age distribution, educational attainment, and even male–female ratios. In addition, some states were more exposed to the opioid crisis due to early marketing strategies for the launch of OxyContin (Alpert et al. 2022), and the demographics of these states are different from less exposed states. This paper implements a simple decomposition framework for thinking about the role of different demographics and exposure to the opioid crisis. Furthermore, given its importance in initiating the opioid crisis, this paper studies the differential impacts of exposure to OxyContin’s launch on overdose rates by demographic group.

The literature has debated whether the overall effects of the opioid crisis are a function of demand (“deaths of despair”) or supply (Maclean et al. 2021; Currie and Schwandt 2021).^2^ This debate is central to understanding the demographic incidence of the opioid crisis. For example, the disproportionate burden shouldered by groups with low education is consistent with reduced economic prospects and limited job opportunities driving demand for substance use (Ho 2017). Yet, as highlighted in Currie and Schwandt (2021), Black Americans have experienced persistently poorer economic outcomes, but have not felt the full force of the opioid crisis when compared to non-Hispanic White Americans until recently.

Due to the severity of the opioid crisis, overdose trends have markedly altered life expectancy (Harper et al. 2021), and there is increasing interest in the evolution of mortality rates across demographic groups. This paper enhances our understanding of broader mortality trends in the United States. The education gradient of mortality has been of special interest to the literature (Halpern-Manners et al. 2020; Everett et al. 2013; Lleras-Muney 2005; Clark and Royer 2013; Novosad et al. 2022; Meghir et al. 2018) as have mortality differences by race and ethnicity (Satcher et al. 2005; Schwandt et al. 2021) and by sex (Geronimus et al. 2019).

I first examine how the opioid crisis has affected overdose death rates for specific demographic groups relative to historical trends. While there are literatures documenting overdose rates for some demographic groups in the context of the opioid crisis, this research tends (with limited exceptions) to examine overdose death rates starting in 1999 or more recently.^3^ By extending these analyses further back, I can study how the opioid crisis has impacted overdose rates across demographic groups relative to historical (i.e., before the opioid crisis, which is often dated as beginning in the mid-1990s) differences. This comparison contextualizes how the opioid crisis has exacerbated pre-existing demographic differences in overdoses as well as which demographic groups have been disproportionately harmed relative to historical rates.

Next, I implement a simple decomposition exercise, which is new in this context, to examine whether some disparities are due to systematic differences in covarying characteristics. For example, overdose rates have historically been higher among men, and this exercise will provide evidence about the extent to which these differences and growth in these differences are associated with sex-specific variation in educational attainment or age. I jointly estimate the overdose time path by race and ethnicity, age, sex, and education to understand which demographic factors are important for determining overdose growth during this time period. This exercise potentially provides insights about whether racial overdose death incidence is partially a function of systematic differences in the age distribution or educational attainment. I extend this exercise to account for differential geographic exposure to the introduction of OxyContin given its importance in igniting the opioid crisis (Alpert et al. 2022; Kolodny et al. 2015; Arteaga and Barone 2022).

Finally, I study heterogeneous responsiveness to OxyContin’s launch using geographic variation discussed in Alpert et al. (2022). Soon after its launch, OxyContin became a blockbuster drug, and Purdue Pharma aggressively marketed it and the use of strong opioids more broadly.^4^ Within just a few years, OxyContin was the most abused opioid in the United States (Cicero et al. 2005). OxyContin was introduced nationwide; however, there is persistent geographic variation in OxyContin supply based on whether a state had a “triplicate prescription program” at the time of OxyContin’s launch. Triplicate programs, which tracked controlled substances from prescriber to pharmacy to a state monitoring agency, were early and especially stringent forms of prescription drug monitoring programs. They led Purdue Pharma to conclude that “The product [OxyContin] should only be positioned to physicians in non-triplicate states...” (Groups Plus 1995). This differential marketing triggered enduring geographic variation in opioid supply, prescribing, and promotional activities. I leverage this variation as a large differential geographic supply shock to understand how OxyContin and its aggressive promotion affected the demographics of overdose death rates over the long term.

This paper contributes three important sets of findings to the literature, which has generally lacked any empirical analyses understanding demographic heterogeneity in overdose death rates beyond documenting recent trends. First, this paper charts the growth of substantial overdose rate disparities across demographic groups and shows when they emerged, extending these trends back to before the beginnings of the opioid crisis. I show how the overdose rate among the Black population increased only modestly during the first wave of the opioid crisis relative to pre-1996 rates while the White and American Indian/Alaska Native (“AI/AN”) populations experienced sharp increases during this same time period. Similarly, there was only modest growth in the male–female overdose disparity through the first wave while the education disparity increased throughout the entire opioid crisis.

Second, I find that some of the most notable observed disparities have actually been *reduced* by covarying characteristics, especially due to systematic differences in educational attainment. For example, non-Hispanic White Americans experienced faster growth than most other racial and ethnic groups for much of the opioid crisis. However, after adjusting for education, age, and sex differences, this gap *increases*, implying that non-Hispanic White Americans have experienced this striking growth despite characteristics which otherwise predict slower growth. Low education groups have experienced the most staggering overdose rate growth since the mid-1990s; yet, the estimated overdose rate disparity between low education and high education groups actually increases further when all other demographic characteristics are held constant. These results suggest that observed disparities often mask even greater adjusted differences. In contrast, men have experienced faster overdose rate growth than women, but this disparity can partially be explained by educational and age differences.

Third, I find that the launch of OxyContin and its lasting effects on opioid access had enduring impacts on overdose death rates across all demographics. Men have been more affected than women by the introduction of OxyContin, though women also experienced large overdose increases in response to increased opioid access, especially relative to historical overdose rates. Individuals with no college education were disproportionately impacted; OxyContin exposure is responsible for a large and growing education disparity in overdose deaths. White, Black, and Hispanic Americans were disproportionately impacted by this supply shock. However, while there is some evidence of differential responses to OxyContin’s launch by race/ethnicity, these differences do not appear to sufficiently explain some of the more notable racial/ethnic disparities in overdose rates.

The analyses suggest that growth in drug overdose rates has been supply-driven across all demographics. These effects continued even after the opioid crisis transitioned to illicit opioid markets, consistent with findings in prior work (Alpert et al. 2018). These results suggest that initial exposure to OxyContin and its enduring effects on opioid access drove large and lasting disparities in overdose death rates across many demographic groups. This finding does not rule out complementary demand-side factors or speak to other possible sources of heterogeneous exposure to the introduction of OxyContin. This shock might itself have been differential across demographic groups even within the same geographic areas. Instead, the results suggest that the introduction and promotion of OxyContin was a critical (though not necessarily sufficient) driver of many observed disparities in overdose death rates.

Understanding the differential impacts of the opioid crisis is important for targeting policies and resources even as it continues to evolve. It is critical to discern the structural causes of these disparities so that appropriate policies can be matched to the underlying reasons for a group’s exposure to the opioid crisis. This paper considers the role of OxyContin and, more generally, medical access to opioids in explaining long-term differential overdose death rates in the US population.^5^

I provide additional background about OxyContin’s launch in the next section. Section 3 describes the data and empirical strategy. I provide results in Sect. 4. I discuss the interpretation of the findings and conclude in Sect. 5.

## Background

### OxyContin’s launch

Recent work concludes that the introduction and marketing of OxyContin played a leading and ongoing role in the growth of overdose death rates (Alpert et al. 2022). OxyContin was introduced to the market in January 1996 by Purdue Pharma. Its key innovation was its long-acting formula which provided 12 h of continuous pain relief, significantly improving the quality and ease of pain management compared with previous drugs. However, the time-release benefit of OxyContin is contingent on taking the pill whole. Crushing or dissolving the pill causes the high dose of oxycodone, intended to be released slowly over 12 hours, to be delivered all at once. This property made OxyContin especially easy to abuse. Purdue Pharma aggressively marketed OxyContin, especially for non-cancer pain (GAO 2003), and it soon became a blockbuster drug with a high rate of misuse.

To understand the initial marketing of OxyContin, I made Freedom of Information Act (FOIA) requests to Florida, Washington, and West Virginia to obtain recently unsealed documents from investigations and court cases brought against Purdue Pharma in these states (see an example in Fig. A.1). These internal Purdue Pharma documents included survey research suggesting that triplicate prescription programs had a chilling effect on the prescribing of strong opioids: physicians were worried about government oversight, and they viewed using the triplicate forms as a major hassle: “Writing triplicate prescriptions was more trouble than others, due to the details of the forms and the various people that need to be copied to them. To the extent that they [physicians] can avoid this extra effort, they will try to follow alternative protocols” (Groups Plus 1995).

The Purdue Pharma documents mention triplicate programs dozens of times, acknowledging that “these regulations create a barrier when positioning OxyContin” (Purdue Pharma 1995). Since there would be lower returns to promoting OxyContin in triplicate states, the internal Purdue Pharma research recommended that “the product [OxyContin] should only be positioned to physicians in non-triplicate states” (Groups Plus 1995).

Triplicate status likely does not represent the only reason for geographic differences in OxyContin promotional activity. The internal Purdue Pharma documents also suggest that there might have been attempts to transition prescribers with a history of prescribing MS Contin to OxyContin, though there is little evidence that this strategy induced notable geographic differences.^6^ There are also mentions of targeting high opioid prescribers, but it is difficult to obtain data to study this dimension specifically. My focus on triplicate status is due to its central importance within the internal Purdue Pharma documents and the substantial geographic variation in opioid access that it induced.

### Triplicate prescription programs

Triplicate prescription programs emerged as some of the first programs to monitor the prescribing and diversion of controlled substances. In a triplicate prescription program, the prescriber is mandated to use state-issued triplicate prescription forms when prescribing Schedule II controlled substances. The prescriber keeps one copy of the prescription. The patient presents the remaining two to the pharmacy, which keeps one copy and sends the other to the state monitoring agency.

At the time of OxyContin’s launch, five states (“triplicate states”) had triplicate programs—California (enacted 1939), Idaho (1967), Illinois (1961), New York (1972), and Texas (1982). Interestingly, these programs were implemented decades before the opioid crisis and were phased out in the years following OxyContin’s launch. Thus, this study examines the longer-term consequences of the *initial* targeting and adoption of OxyContin. I use the 1996 launch of OxyContin as a large, differential shock to opioid access to study its long-term consequences on overdose disparities.^7^

Figure A.2 displays evidence across multiple available data sources that non-triplicate states were more “exposed,” in terms of supply and promotional activity, to the introduction of OxyContin. Panel A shows promotional payments for OxyContin, obtained from the Open Payments data base for August 2013 through the end of 2016 (Centers for Medicare and Medicaid Services 2018).^8^ Promotional activities for OxyContin differ substantially between triplicate and non-triplicate states. It might be surprising that triplicate status in 1996 could have such long-term effects on marketing strategies, but a defining feature of Purdue Pharma’s detailing strategy was its persistence. Internal documents suggest that a core strategy of the sales force was to call and visit the top OxyContin prescribers, and this behavior continued, and became even more frequent, through 2018.^9^ Thus, contemporary differences in promotional activities reflect variation in initial targeting, amplified by a marketing strategy that targeted high prescribers.

I also examine early and longer-term differences in OxyContin “adoption” as measured in terms of prescriptions and morphine equivalent doses. I provide differences in per capita OxyContin morphine equivalent doses from the Automation of Reports and Consolidated Orders System (ARCOS) data (Drug Enforcement Agency 2018) for 2000–2016 in Panel B,^10^ per beneficiary Medicaid prescriptions from the State Drug Utilization Data (SDUD) data (Centers for Medicare and Medicaid Services 2015) for 1996–2005 in Panel C,^11^ and per capita prescriptions in the Medical Expenditure Panel Survey (MEPS) (Agency for Healthcare Research and Quality 2018) for 1996–2016 in Panel D.^12^ In all cases, triplicate states have substantially less access and use of OxyContin, both in the years immediately after the launch in 1996 and continuing through the most recent years of data.^13^ Moreover, there were substantial spillovers to other strong oxycodone products because Purdue Pharma promoted the use of oxycodone more broadly.^14^ Thus, total oxycodone (and opioid supply) differences between non-triplicate and triplicate states exceed the differences in OxyContin (see Fig. A.3).

## Data and empirical analysis

### Data

I use the restricted geocoded National Vital Statistics System (NVSS) Multiple Cause of Death mortality files—the census of deaths in the United States—to study annual overdose deaths from 1989 to 2020 (Centers for Disease Control and Prevention 2020). I begin the sample period in 1989 because the 1989 revision provided new categorizations of educational attainment, which was immediately adopted by almost all states. In addition, 1989 was the first year that the Standard Certificate of Death included the Hispanic origin of the decedent.^15^ Because of this paper’s interest in education disparities, I study overdose deaths for ages 25+.

For 1989–1998, I define drug poisonings as deaths involving underlying cause of death ICD-9 codes E850-E858, E950.0-E950.5, E962.0, or E980.0-E980.5, following the CDC.^16^ For the 1999–2020 data, I code deaths as drug overdoses using the ICD-10 external cause of injury codes X40-X44, X60–64, X85, or Y10-Y14 (Warner et al. 2011).^17^ I focus on all overdose deaths, regardless of substances involved, to understand how the demographics of fatal drug overdoses have changed over time; using all overdoses also provides improved consistency over this period.^18^ I show supplementary results for overdoses involving opioids.

The death certificates include demographic information, although the consistency and inclusion of these variables can be limited. For non-Hispanic decedents, I use the following “bridged race” groups as categorized in the NVSS codebooks: “American Indian (includes Aleuts and Eskimos)” (which I will refer to as “AI/ANs”),^19^ an aggregation of multiple categories for Asian-Americans and Pacific Islanders (“AAPIs”),^20^ “Black,” and “White.” I examine Hispanic Americans separately; thus, I study five non-overlapping racial and ethnic groups.^21^

Education is reported in the NVSS data beginning in 1989; however, the education coding changed due to a 2003 revision which was not adopted universally so the old coding is sometimes used in subsequent years until 2018. I construct two education categories which should be relatively consistent across this coding change: “no college” and “college,” defined as having attended one or more years of college. This coding is also used in Case and Deaton (2017).^22^ Georgia, Oklahoma, Rhode Island, and South Dakota were slow to add the education item (Rostron et al. 2010) so I drop these states from the analysis. A small percentage (4.0%) of the remaining death certificates in the sample do not include a known education level. I assign these deaths the same education distribution as the certificates from overdoses with education reported with the same state, year, race, ethnicity, sex, and age group.^23^

To construct rates, I scale by population size using Medicare SEER population data.^24^ These data do not include education information, so I construct education shares from the Current Population Study to impute population size by education status.^25^

### Empirical analysis

#### Quantifying historical trends

I evaluate overdose trends by race and ethnicity, sex, and education. For each set of analyses, I first show overdose death rates stratified by demographics to illustrate variation in the impact of the opioid crisis. While the literature has documented some of this heterogeneity for overdoses, it presents this information beginning in 1999 or, typically, even more recently. Truncating the data at 1999 or later limits our ability to inspect how relative demographic trends evolved and potentially flipped due to the opioid crisis. Moreover, less attention has been paid in the opioid crisis literature to the historical trends of some demographic groups, such as the AI/AN population,^26^ who have been disproportionately affected by the growth in overdose death rates. I provide these time series analyses to understand the emergence of these disparities. These descriptive analyses are a contribution of this paper.

I also study the evolution of these trends while accounting jointly for other demographic and geographic factors. Let $y_{rgcast}$ represent the overdose death rate of racial/ethnic group *r*, sex *g*, educational attainment *c*, and age group (25–34, 35–44, 45–54, 55–64, 65–74, 75+) *a* in state *s* at time *t*. I present descriptive results from the following model:

1 $$\begin{aligned} y_{rgcast} = \alpha _{rt} + \beta _{gt}+ \gamma _{at} + \delta _{ct} + \text{ Non-Triplicate}_s \tau _t + \epsilon _{rgcast}. \end{aligned}$$

The specification includes race/ethnicity $\times$ time interactions, sex $\times$ time interactions, age group $\times$ time interaction, education $\times$ time interactions, and Non-Triplicate $\times$ time interactions. It relies on the fact that while there are correlations in demographics (as documented above), there remains substantial overlap across categories. No demographic characteristic perfectly predicts another. The specification considers whether and to what extent the high overdose rate experienced by a racial/ethnic group is shared across education categories, age groups, sex, and geography or, otherwise, specific to a certain demographic factor across all races and ethnicities. This decomposition exercise explores whether some of the disproportionate increase in overdose rates among specific racial/ethnic populations can be explained by differences in age distributions (for example) across racial/ethnic groups. If similar increases in overdose rates are observed across all racial/ethnic groups for a particular age group, this analysis attributes those changes to age-related factors rather than race/ethnicity.

Presenting estimates relative to an excluded category would make comparisons across the non-excluded groups difficult, so I present estimates relative to the average. As an example, the overdose mortality rate of AI/AN individuals is presented relative to the average rate across all racial/ethnic groups, including AI/AN individuals themselves.^27^ With pairwise comparisons (i.e., when studying mortality rates by sex or by education), constructing predictions relative to the mean is less necessary, but I remain consistent with the race/ethnicity results and report results relative to the average.

For each demographic group, I present three sets of estimates. First, I present the unadjusted rates, which are equivalent to the population-weighted averages of the predicted values from Eq. 1. Second, I present population-weighted predictions adjusted for demographics, which are the predictions from Eq. 1 that exclude the estimates of the other demographic factors (i.e., holding other demographics constant). Finally, I include rates adjusted for demographics *and* initial triplicate status, which are differences in population-weighted predictions that exclude the estimates of the other demographics and from the triplicate variable (i.e., just using the $\alpha _{rt}$ estimates when studying race/ethnicity). I discuss the motivation for exploring the importance of these adjustments in Sect. 3.2.3.

I provide year-by-year estimates from these models, but I also aggregate them for 1996–2000, 2001–2010, 2011–2020, and the full post-period 1996–2020. The choice of these time periods is discussed more in the next section. When presenting these aggregated differentials, I subtract off the 1991–1995 predictions as the baseline differences. The results then can be interpreted as the additional *growth* in overdose death rates for a specific demographic group relative to the mean growth across all demographic groups for that time period. For the corresponding figures, I do not subtract off the 1991–1995 baseline.

These models are descriptive as they do not provide insight on whether low education groups experienced sharper growth because of their education or due to other (excluded) factors that correlate with education (e.g., income, marital status). However, this decomposition exercise provides useful information about which disparities appear to be driven by covarying factors.^28^ I compare the unadjusted and adjusted trends to help understand whether overdose trends vary with education or because education correlates with other demographic characteristics. I use demographic-specific population weights when estimating Eq. 1. Standard errors are adjusted for state-level dependence.

#### The role of OxyContin

I consider OxyContin’s role in explaining overdose death disparities in two ways. The above analysis will provide evidence about a “mechanical effect” of overdose rate disparities due to the geography of Purdue Pharma’s promotional activity and differences in demographics in these areas. Triplicate states and non-triplicate states experienced very different exposure to OxyContin’s launch. These states have different demographics such that, mechanically, some demographic groups were more exposed to the launch than others due solely to having larger population shares in non-triplicate states. I estimate this mechanical effect. This approach assumes a fixed response to OxyContin’s launch across demographic groups.

In a complementary analysis, I estimate how demographic groups *differentially* responded to OxyContin exposure, using geographic variation in its initial targeting. Triplicate states were less exposed to OxyContin’s launch and the opioid crisis more broadly, providing evidence of how overdose rate disparities across demographics might have evolved in the (partial) absence of OxyContin.^29^ The non-triplicate states provide evidence about how additional exposure to the opioid crisis altered these differences. This analysis shows how different demographic groups responded to the OxyContin’s introduction and promotion.

I implement a difference-in-differences design, comparing changes in overdose rates in non-triplicate states (more exposed) to changes in triplicate states (less exposed). I estimate the following specification by demographic group:

2 $$\begin{aligned} y_{st} = \alpha _s + \gamma _t +\beta _t \textbf{1}\!\left( \text{ Non-Triplicate}_s\right) + \epsilon _{st}, \end{aligned}$$

where $y_{st}$ is overdoses per 100,000 people in state *s* and year *t* for the population of interest. This “event study” specification includes state and time fixed effects while permitting differential annual growth in the overdose rate for non-triplicate states, indexing the coefficient by *t*.^30^ I normalize $\beta _{1995}=0$. $\beta _t$ represents the differential overdose rate growth in non-triplicate states in year *t* (relative to 1995) and can be interpreted as the response resulting from additional exposure to OxyContin’s launch. Non-triplicate status is fixed to its value in 1996, the year of OxyContin’s launch, so these estimates refer to the differential annual growth due to *initial* non-triplicate status.

The pre-1996 coefficient estimates provide evidence about the existence or non-existence of trends prior to OxyContin’s launch. There is little evidence of systematic pre-1996 movements between non-triplicate and triplicate states, consistent with findings in Alpert et al. (2022).^31^

To summarize the event study results, I present averages of the $\beta _t$ estimates by demographic group relative to 1991–1995.^32^ I provide these aggregates for 1996–2000, representing the introduction of OxyContin and ramp up of marketing by Purdue Pharma; 2001–2010, representing the “first wave” of the opioid crisis; and 2011–2020, representing the subsequent (“illicit”) waves of the opioid crisis. I expect early OxyContin exposure to potentially have effects even as the opioid crisis later transitioned to illicit opioids given that previous research has shown that reformulation, through geographic exposure to OxyContin, induced the transition to heroin (Alpert et al. 2018) and, later, to fentanyl (Powell and Pacula 2021), increasing overdose rates.^33^ I also include averages for the entire 1996–2020 post-period.

I estimate Eq. 2 using weighted (by demographic population size) least squares. For consistency with the Sect. 3.2.1 analysis, I exclude the four states with incomplete education information. Due to the small number of triplicate states, traditional clustered (by state) standard errors would likely lead to over-rejection (Cameron et al. 2008).^34^ Instead, I use a clustered (by state) wild bootstrap with Webb (2023) weights to construct 95% confidence intervals (Roodman et al. 2018). This approach tests a series of null hypotheses and constructs confidence intervals based on the hypotheses that are not rejected. Given this process, the confidence intervals are not symmetric.

#### Summary statistics of covarying demographic and geographic characteristics

The motivation for considering covarying demographic characteristics is that there are notable differences in education rates, age, and even male–female ratios across racial and ethnic groups. These summary statistics are presented in Table A.1 for the baseline period 1991–1995 and for 1996–2000, 2001–2010, 2011–2020, and 1996–2020. *Changes* in demographics are likely less important here than the levels. If overdose rate growth primarily affected individuals with no college education, then cross-sectional variation in educational attainment rates is the important factor, and it matters less whether there was a rise in the share not attending college. White and AAPI individuals tend to have higher education levels which, as discussed below, is correlated with slower overdose growth. White Americans tend to be older which also predicts slower overdose rate growth. There are even large differences in sex ratios across racial and ethnic groups.

Table A.2 provides demographic information by sex. There are notable differences in the age distributions—22.7% of females are aged 65+ compared to only 18.4% of males (for the full 1996–2020 time period). The educational attainment rates also vary in some time periods. For 2011–2022, 41.3% of males have not attended any college compared to 38.3% of females. Table A.3 includes the equivalent information stratified by education. The age distributions between the “no college” and “some college” groups are notably different as those with at least some college experience tend to be younger.

Finally, the motivation for considering systematic variation in residence in a triplicate state is due to differences in the demographics of these states. I show these differences in Table A.4. Panels A and B shows that educational attainment rates and male–female ratios are similar across triplicate and non-triplicate states. However, there are large racial and ethnic differences (see Panels C–G), suggesting that there is some potential that the low overdose rate growth for AAPI and/or Hispanic individuals is due to their higher rates of living in triplicate states. The exercise discussed in Sect. 3.2.1 will evaluate the empirical importance of these geographic differences.

**Fig. 1:**
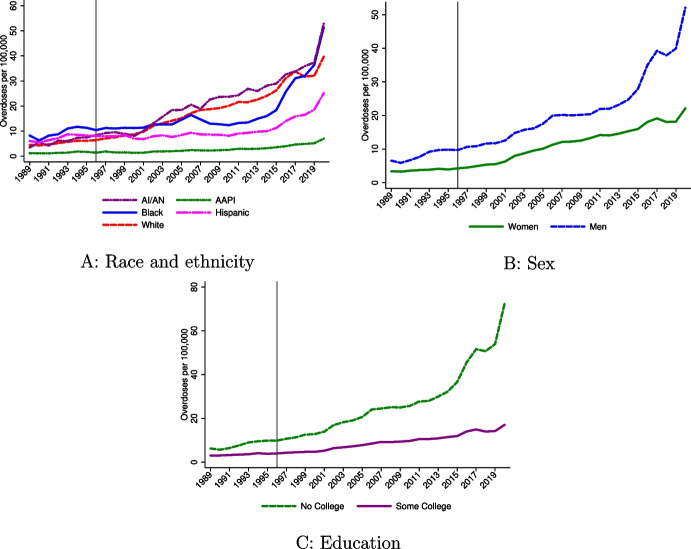
Drug overdose death rates for 1989–2020 by demographic group. Notes: I plot (unadjusted) drug overdose death rates for 1989–2020 using NVSS data for ages 25+. For 1989–1998, I define drug poisonings as deaths involving underlying cause of death ICD-9 codes E850-E858, E950.0-E950.5, E962.0, or E980.0-E980.5. For the 1999–2020 data, I code deaths as drug overdoses using the ICD-10 external cause of injury codes X40-X44, X60-64, X85, or Y10-Y14. Ethnicity and education information is only available beginning in 1989. “Some college” means at least some college education. Overdose deaths are scaled by population using Census, SEER, and CPS data (see text for details). For **C** only, Georgia, Oklahoma, Rhode Island, and South Dakota are excluded because of missing education information early in the sample period. See Fig. A.4 for replications of **A** and **B** without these four states

## Results

### Understanding overdose death rate trends

#### Overdose rates by race and ethnicity

Figure 1 shows trends in overdose death rates by demographic group. In Panel A, I present unadjusted overdose death rates by race and ethnicity for ages 25+: Hispanic Americans and Non-Hispanic AI/AN, AAPI, Black, and White individuals. I include all states here to show national trends. In Appendix Fig. A.4, I provide the same time series but excluding the four states with missing education information (i.e., the analysis sample for much of this paper). Overdose death rates were highest for Black Americans before 1996, but White and AI/AN Americans experienced the sharpest growth since OxyContin’s launch. The Black population had relatively flat overdose death rate growth through the first two waves of the opioid crisis and appeared to be generally less affected by the opioid epidemic until the fentanyl crisis, beginning in 2014. From 1995 to 2009, the overdose death rate among the Black population (ages 25+) increased by 1.4/100,000, exhibiting substantially less growth than the 13.0/100,000 additional overdose deaths among the White population and the 16.2/100,000 additional deaths among the AI/AN population.^35^ In more recent years, Black individuals have incurred staggering overdose rate growth. The Hispanic population had high overdose deaths prior to 1996; however, they experienced relatively slow growth in overdose deaths until around the time of the fentanyl crisis. Their growth since 2014 has been more modest than non-Hispanic populations. The AAPI population had lower levels of overdose rate growth through the entire sample period.

**Table 1:** Decomposition results by race and ethnicity

Period	(1)	(2)	(3)
Panel A: AI/AN			
1996–2000	0.662	0.288	0.184
	[−0.481, 1.806]	[−0.884, 1.460]	[−0.989, 1.357]
2001–2010	3.346**	1.906	1.625
	[0.471, 6.221]	[−1.137, 4.950]	[−1.473, 4.723]
2011–2020	7.153**	3.549	2.754
	[0.671, 13.635]	[−3.350, 10.449]	[−4.311, 9.819]
1996–2020	4.831**	2.548	2.008
	[0.929, 8.734]	[−1.611, 6.707]	[−2.264, 6.280]
Panel B: AAPI			
1996–2000	−1.684***	−1.423***	−1.113***
	[−2.303, −1.065]	[−2.059, −0.786]	[−1.758, −0.468]
2001–2010	−7.203***	−6.226***	−5.336***
	[−9.021, −5.386]	[−8.058, −4.394]	[−7.233, −3.438]
2011–2020	−15.847***	−14.320***	−12.661***
	[−19.538, −12.157]	[−18.042, −10.597]	[−16.475, −8.846]
1996–2020	−9.776***	−8.862***	−7.941***
	[−12.219, −7.332]	[−11.375, −6.350]	[−10.484, −5.397]
Panel C: Black			
1996–2000	−0.803	−1.032**	−1.043**
	[−1.865, 0.258]	[−2.033, −0.031]	[−2.048, −0.037]
2001–2010	−4.679***	−5.996***	−6.041***
	[−6.361, −2.996]	[−7.750, −4.241]	[−7.843, −4.240]
2011–2020	−2.212	−5.216***	−5.451***
	[−5.909, 1.484]	[−9.009, −1.423]	[−9.272, −1.630]
1996–2020	−2.694**	−4.632***	−4.813***
	[−5.077, −0.310]	[−7.066, −2.198]	[−7.272, −2.354]
Panel D: Hispanic			
1996–2000	−1.638***	−2.567***	−2.143***
	[−2.199, −1.078]	[−3.091, −2.042]	[−2.593, −1.694]
2001–2010	−7.379***	−11.325***	−10.172***
	[−8.544, −6.215]	[−12.726, −9.925]	[−11.470, −8.873]
2011–2020	−12.057***	−22.186***	−20.083***
	[−13.846, −10.268]	[−25.208, −19.164]	[−23.086, −17.081]
1996–2020	−7.954***	−14.828***	−13.552***
	[−9.213, −6.696]	[−16.692, −12.964]	[−15.373, −11.731]
Panel E: White			
1996–2000	0.375***	0.571***	0.533***
	[0.216, 0.534]	[0.425, 0.716]	[0.380, 0.686]
2001–2010	2.382***	3.368***	3.204***
	[1.992, 2.772]	[2.883, 3.853]	[2.736, 3.671]
2011–2020	4.380***	7.372***	6.947***
	[3.592, 5.168]	[6.237, 8.506]	[5.867, 8.027]
1996–2020	2.512***	4.243***	4.074***
	[2.038, 2.985]	[3.628, 4.858]	[3.489, 4.660]

Notes: Outcome is all drug overdose deaths per 100,000 for the listed demographic. I report predictions relative to 1991–1995 and relative to the mean change in overdose deaths over all demographic groups. Column 1 reports the unadjusted rates relative to the mean. Column 2 holds other demographics (age, sex, and education) constant. Column 3 adjusts for other demographics and 1996 triplicate status. 95% confidence intervals reported in brackets are adjusted for state-level dependence. *$p < 0.1$; **$p < 0.05$; ***$p < 0.01$

I summarize these trends in Table 1, which provides the overdose death rate in Column 1 for the listed period relative to the 1991–1995 rate for each racial/ethnic group compared to the average growth.^36^ I provide averages (relative to 1991–1995) for 1996–2000, 2001–2010, and 2011–2020 plus an aggregate for the full post-period 1996–2020. To see annual estimates for the full 1989–2020 period, refer to Fig. A.5. The AI/AN population experienced the sharpest increase for each of the time periods relative to the baseline period, followed by non-Hispanic White individuals. Over 1996–2020, AI/AN individuals experienced an additional 4.8/100,000 annual overdose deaths compared to the average. In contrast, White individuals experienced an additional 2.5/100,000 while Black Americans experienced a reduction of 2.7/100,000 compared to the mean. The Hispanic and AAPI populations experienced very large relative (to the mean) reductions: 8.0/100,000 and 9.8/100,000 per year, on average, respectively. The magnitudes of these disparities generally grew over time.^37^

In Column 2 of Table 1 (and see Fig. A.5), I present the same estimates but adjusted for variation in age, education, and sex. Demographic differences (discussed above in Table A.1) appear to be important. The high overdose death rate disparity for the AI/AN population declines by 47% for the full 1996–2020 period when age, education, and sex are held constant. I also find that some (about 9%) of the lower overdose growth experienced by AAPI individuals can be attributed to other factors such as higher education rates. On the other hand, when accounting for other demographics, the estimated disparities for the Black and Hispanic populations become even more negative. These changes are especially notable for the 2011–2020 time period—the estimates for Black individuals increase in magnitude by 136% and for Hispanic individuals by 84% when adjusted for differences in age, education, and sex. White individuals experienced relatively high overdose death rate, but this disparity increases even further when other factors are considered (by 69% for the full 1996–2020 period). These results generally suggest that some of the more notable racial and ethnic overdose disparities are actually *attenuated* due to covarying protective factors.

Table A.5 offers a more detailed breakdown of the demographic characteristics that have the greatest impact. In this analysis, only one demographic category—age, sex, or education—is held constant at a time. For each racial/ethnic group, education emerges as the most influential factor, resulting in the largest change to the 1996–2020 estimate when held constant. In some cases, educational attainment differences contribute to overdose disparities. However, in many cases, they actually reduce them. For example, the differential incidence of overdose deaths among the White population would be even larger given educational attainment rates comparable to the full population.

In Column 3 of Table 1, I adjust for initial triplicate status to consider the effects of differential exposure to the early marketing of Purdue Pharma. Also, see Fig. A.5 for annual estimates. The estimated disparities are meaningfully impacted though less so when compared to the effects of the demographic adjustments. Part of the higher overdose rates incurred by the AI/AN population results from disproportionately residing in non-triplicate states. The conditional disparity decreases by an additional 21% when this factor is accounted for (comparing Column 3 to Column 2). The AAPI population has higher shares in triplicate states and this explain 12% of the disparity estimated in Column 2. Similarly, the Hispanic population partially (9% comparing Column 3 to Column 2) incurred lower overdose rates because they were more likely to live in triplicate states. The relative lack of exposure to the early marketing of Purdue Pharma represented by initial triplicate status explains about 4% of the lower overdose rates for Black Americans.^38^

Overall, the role of geography appears less important, possibly because this analysis only considers the effects of triplicate status, an impactful but coarse designation. While triplicate status itself has a large effect and some of the demographic differences across triplicate and non-triplicate states are vast,^39^ the interaction of these two quantities—when a common effect across all demographic groups is enforced—cannot explain more meaningful national demographic heterogeneity in overdose death rate growth.

**Table 2:** Decomposition results by sex and educational attainment

Period	(1)	(2)	(3)
Panel A: Male			
1996–2000	0.623***	0.547***	0.550***
	[0.287, 0.959]	[0.214, 0.880]	[0.217, 0.883]
2001–2010	1.284***	0.864**	0.872**
	[0.438, 2.130]	[0.057, 1.671]	[0.068, 1.676]
2011–2020	5.637***	4.442***	4.447***
	[4.116, 7.159]	[3.125, 5.758]	[3.133, 5.762]
1996–2020	3.117***	2.396***	2.390***
	[2.125, 4.109]	[1.505, 3.287]	[1.501, 3.280]
Panel B: No college			
1996–2000	1.322***	1.602***	1.587***
	[1.006, 1.638]	[1.209, 1.994]	[1.201, 1.972]
2001–2010	5.031***	6.436***	6.380***
	[4.081, 5.981]	[5.217, 7.654]	[5.147, 7.614]
2011–2020	15.548***	18.391***	18.421***
	[12.523, 18.573]	[14.866, 21.917]	[14.887, 21.955]
1996–2020	7.817***	9.657***	9.794***
	[6.344, 9.290]	[7.957, 11.356]	[8.083, 11.504]

Notes: Outcome is all drug overdose deaths per 100,000 for the listed demographic. I report predictions relative to 1991–1995 and relative to the mean change in overdose deaths over all demographic groups. Column 1 reports the unadjusted rates relative to the mean. Column 2 holds other demographics (age, race/ethnicity, and either education or sex) constant. Column 3 adjusts for other demographics and 1996 triplicate status. 95% confidence intervals reported in brackets are adjusted for state-level dependence. *$p < 0.1$; **$p < 0.05$; ***$p < 0.01$

#### Overdose rates by sex

Figure 1B shows overdose death trends by sex (see Fig. A.4 for the same trends but excluding the four states without education information). The overdose death rate for women was flat prior to 1996, before steadily increasing since. Men experienced a sharp and steady increase in overdose death rates that deviates from the pre-1996 trend. Relative to other demographic factors, the disparity in unadjusted overdose death rate growth is less stark between males and females through the first wave. Between 1995 and 2009, females experienced an increase in overdose deaths of 8.6/100,000 while the male overdose rate grew by 10.4/100,000 over the same time period (Fig. 1B). However, in more recent years, men have experienced staggering growth in overdose deaths: 31.9/100,000 additional annual deaths in 2020 relative to 2009 compared to 9.6/100,000 for women.

I summarize these differences in Table 2, Panel A. The equivalent year-by-year point estimates are provided in Fig. A.6. Men experienced 3.1/100,000 additional overdose deaths per year relative to the average during the full 1996–2020 period. This implies that the male–female disparity in overdose rates was, on average, 6.2/100,000 deaths higher in each year since 1996 (relative to 1991–1995). However, one-quarter of this growth is not attributed specifically to sex but to covarying demographic differences (see Column 2). The disparity estimate is relatively unaffected by accounting for geography (Column 3) given that the male–female shares are similar across triplicate and non-triplicate states.

Table A.6 Panel A provides a more detailed assessment of the importance of the demographic variables. As before, education is the most important factor. Educational differences alone explain about 12% of the male–female disparity in overdose deaths, primarily due to the higher education rates of women in 2011–2020.

#### Overdose rates by education

The changing education gradient of mortality is a central theme of the “deaths of despair” analysis. There have been substantial differences in overdose rate growth between the “no college” and “some college” population, as shown in Fig. 1C, over the entire post-1996 time period. For example, from 2009 to 2020, the overdose rate among those without any college experience increased by 47.4/100,000 compared to 7.7/100,000 for those with at least some college experience. As Table 2 (Panel B) shows (also see Fig. A.6, Panel B), education is predictive of especially large disparities. Over 1996-2020, individuals with no college education experienced higher overdose rates growth of 7.8 per 100,000, relative to the mean and to the baseline period, with especially large growth in more recent years. Because of how these estimates are reported, this estimate implies that the difference in overdose death rates between the “no college” and “some college” groups was 15.6/100,000 higher on average than the baseline period. This rate actually increases by 24% when adjusted for other characteristics (Column 2), further suggesting that education is an especially important predictor of exposure to the opioid crisis. Accounting for geographic factors has much less impact on the estimated education disparity.

Appendix Table A.6 Panel B considers the relative importance of each of the demographic variables. In this case, adjusting for race/ethnicity differences increases the estimated education disparity by an additional 17%.

**Fig. 2:**
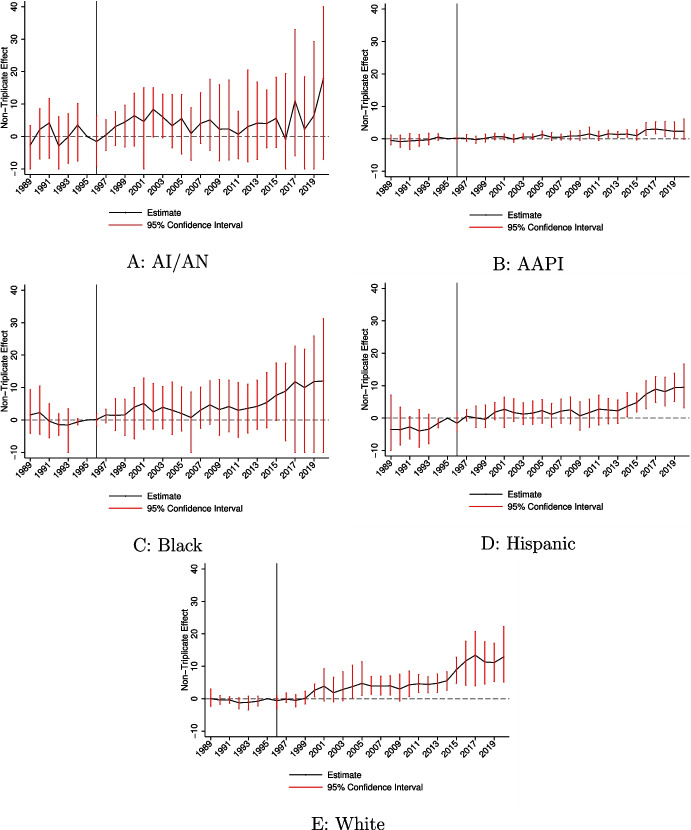
Event study estimates comparing non-triplicate to triplicate states by race and ethnicity. Notes: **A**–**E** provide event study estimates from estimation of Eq. 2 in the paper. The model includes state and year fixed effects. The estimates represent the differential change in the overdose rate in non-triplicate states. The 1995 coefficient is normalized to zero. Regressions are weighted by the population size for the demographic group. 95% confidence intervals are estimated using a clustered (by state) wild bootstrap. Confidence intervals are truncated at −10 and 40 (in rare cases) to improve readability of the figures

**Table 3:** Difference-in-differences estimates by demographic group comparing non-triplicate states to triplicate states

Panel A: Race and ethnicity					
Period	AI/AN	AAPI	Black	Hispanic	White
1996–2000	1.635	0.370	2.523*	2.452***	1.000**
	[−0.814, 5.005]	[−0.283, 0.805]	[−0.439, 5.403]	[0.814, 4.463]	[0.245, 1.970]
2001–2010	3.311	0.906	4.023	4.127**	4.312**
	[−1.877, 11.417]	[−0.479, 2.641]	[−0.707, 10.709]	[0.386, 7.446]	[1.143, 7.607]
2011–2020	4.466	2.105**	8.625	8.275***	9.577***
	[−6.776, 21.076]	[0.055, 5.247]	[−3.862, 16.826]	[4.635, 12.166]	[5.098, 13.364]
1996–2020	3.438	1.278*	5.564*	5.451***	5.756***
	[−2.977, 12.748]	[−0.187, 3.340]	[−0.759, 11.770]	[2.486, 8.488]	[3.747, 7.493]
Panel B: Sex					
Period	Men	Women			
1996–2000	2.573***	0.697**			
	[1.058, 3.748]	[0.151, 1.356]			
2001–2010	7.970***	3.711***			
	[3.244, 11.222]	[1.927, 5.859]			
2011–2020	15.954***	8.435***			
	[6.610, 22.194]	[5.044, 10.585]			
1996–2020	10.084***	4.998***			
	[4.846, 13.528]	[3.456, 6.083]			
Panel C: Education					
Period	No college	Some college			
1996–2000	2.231**	0.642*			
	[0.580, 4.431]	[–0.001, 1.109]			
2001–2010	9.483***	2.057***			
	[4.613, 13.438]	[1.020, 2.810]			
2011–2020	23.153***	4.248***			
	[9.391, 30.934]	[1.350, 6.052]			
1996–2020	13.501***	2.650***			
	[5.889, 17.165]	[1.333, 3.515]			

Notes: Outcome is all drug overdose deaths per 100,000 for the listed demographic. I estimate an event study, conditioning on state and year fixed effects. The event study estimates refer to the additional overdoses experienced in non-triplicate states by year. I average these estimates for the years listed in the first column. Estimates are relative to the pre-period, 1991–1995. 95% confidence intervals reported in brackets are estimated by clustered (by state) wild bootstrap. * $p < 0.1$; ** $p < 0.05$; *** $p < 0.01$

#### Discussion of decomposition findings

This analysis revealed that some observed disparities in overdose death rates are partially influenced by covarying demographic factors. For instance, while the AI/AN population has experienced disproportionately high overdose death rates, a significant portion of this can be attributed to other demographic characteristics. Similarly, males have higher overdose death rates than females, but some of this disparity is explained by other demographic differences.

Interestingly, many racial and ethnic disparities between groups appear larger once adjustments for age, sex, and education are made. For example, Hispanic Americans have consistently had low overdose death rates throughout the opioid crisis, but these rates are even lower than would be expected based on their demographic profile. Similarly, the Black population experienced slower growth in overdose death rates for much of the crisis than expected given their age distribution and educational attainment levels. In contrast, the White population has experienced overdose death rates that are higher than would be predicted by their age, sex, and education distribution.

Geography also played a meaningful role, albeit to a lesser extent than demographics. The analysis relied on a coarse geographic level given evidence of differential marketing practices by Purdue Pharma across states. However, it is likely that promotional activities varied in more nuanced ways not captured in the unsealed documents used for this study, so I cannot rule out that geography played a more substantive, unobserved role.

**Fig. 3:**
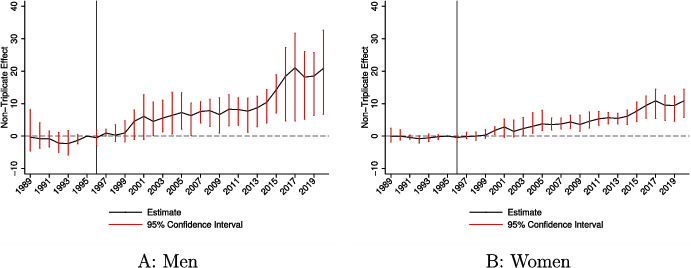
Event study estimates comparing non-triplicate to triplicate states by sex. Notes: **A** and **B** provide event study estimates from estimation of Eq. 2 in the paper. The model includes state and year fixed effects. The estimates represent the differential change in the overdose rate in non-triplicate states. The 1995 coefficient is normalized to zero. Regressions are weighted by the population size for the demographic group. 95% confidence intervals are estimated using a clustered (by state) wild bootstrap

### Differential responses to OxyContin’s introduction

The above analysis finds a role for the differential geography of OxyContin’s promotion at launch for explaining national disparities, holding constant the impact of OxyContin. In this section, I test for differential *responses* to OxyContin’s launch by comparing states more exposed to its launch to less exposed states.

Estimating Eq. 2, I present event study results by race and ethnicity in Fig. 2, and I summarize the estimates in Table 3. I find some evidence of heterogeneity across racial and ethnic groups. Overall, effect sizes tend to increase over time across all demographic groups, even after the original formulation of OxyContin was withdrawn from the market. This pattern aligns with findings in the broader literature on the general population (Powell and Pacula 2021). The largest effects are estimated for non-Hispanic White individuals (Panel E). I also estimate large effects for the Black (Panel C) and Hispanic (Panel D) populations, though the estimates for the Black population are less precise. The AI/AN population experienced more modest increases in overdose death rates due to the introduction of OxyContin (Panel A), though these estimates are much less precisely-estimated. The AAPI population incurred the smallest overdose rate growth in response to OxyContin’s introduction (Panel B).

The magnitude of the relationship between exposure to OxyContin and overdose deaths among the Black and Hispanic populations are especially notable given their lower rates of growth nationally for much of the post-1996 period. These results suggest that these populations experienced especially low baseline rates of growth in triplicate states. We discuss this issue more in the next section.

I observe especially sharp growth in the long-run impact of OxyContin’s launch and promotion for most racial and ethnic populations once the fentanyl crisis begins. Exposure to OxyContin predicts the transition to illicit markets after reformulation, which caused illicit opioid markets to grow, eventually causing fentanyl to bleed into other drug markets. Thus, the rate of overdose deaths for populations that had generally been less directly affected by the opioid crisis began to increase as fentanyl permeated other illicit drug supplies.

Across all demographic groups, I typically observe stronger evidence of effects when focusing on overdoses involving opioids (see Table A.7). It is likely that the measure of opioid-related overdoses is missing some relevant deaths due to misclassification, suggesting that the point estimates should generally be smaller. However, it is not surprising that this analysis generally provides more precise evidence given that it excludes many deaths that are likely to be unrelated to the opioid crisis.

Figure 3 provides event study estimates by sex. I estimate large effects for both men and women, though the effect sizes are substantially larger for men. Over the full 1996–2020 post-period, additional exposure to OxyContin’s launch induced 10.1 overdose deaths per 100,000 men compared to 5.0 for women (see Table 3). In every period, men were more affected by the introduction of OxyContin. While women were meaningfully impacted, OxyContin’s launch and the differential response by sex appear to explain growing mortality disparities.

**Fig. 4:**
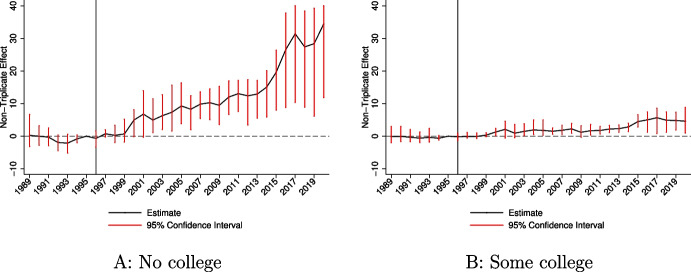
Event study estimates comparing non-triplicate to triplicate states by education. Notes: **A** and **B** provide event study estimates from estimation of Eq. 2 in the paper. The model includes state and year fixed effects. The estimates represent the differential change in the overdose rate in non-triplicate states. The 1995 coefficient is normalized to zero. Regressions are weighted by the population size for the demographic group. 95% confidence intervals are estimated using a clustered (by state) wild bootstrap. Confidence intervals are truncated at −10 and 40 to improve readability of the figures

I study education differences in Fig. 4. I observe much larger effects for the population without any college education. Over the 1996–2020 period, the additional exposure to OxyContin’s introduction due to non-triplicate status increased annual overdose rates by 13.5 deaths per 100,000 for those with no college education, compared to 2.7 for those with some college education or more (see Table 3). The disparities generated during the illicit opioid crisis are particularly striking, with estimated effects of 23.2/100,000 compared to 4.2/100,000, highlighting the significant predictive power of educational attainment during this period, as discussed in Powell (2023). These findings indicate that education has been a particularly influential factor in determining who was most affected by OxyContin and its downstream consequences. This differential response generated substantial changes to the education gap in overdose death rates and mortality rates more generally. Again, see Table A.7 for opioid-specific results.

**Fig. 5:**
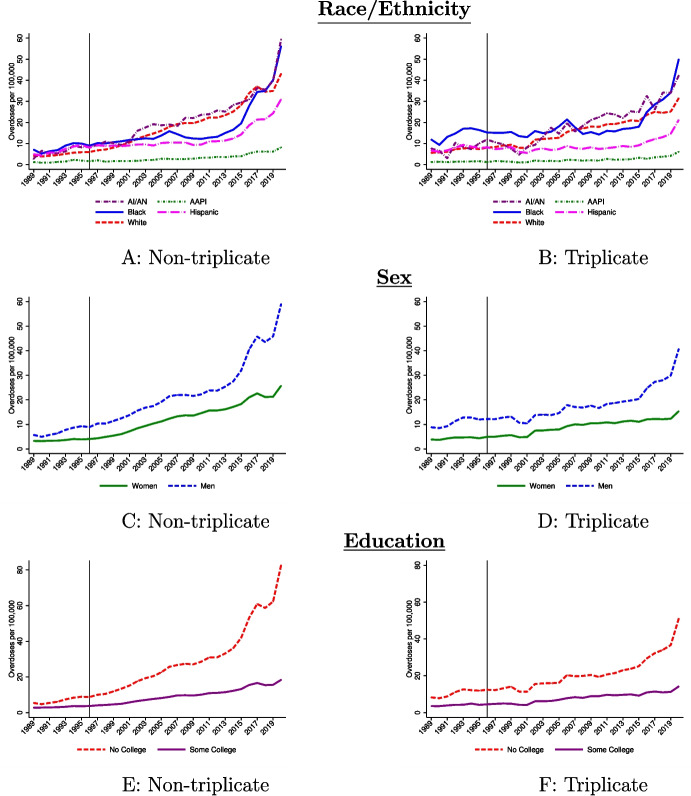
Drug overdose death rates by triplicate status and demographic group. Notes: These figures compare trends in overdose deaths for 100,000 by demographics for non-triplicate and triplicate states (excluding the four states with incomplete education information)

### Discussion of results

The launch of OxyContin led to significant disparities in overdose death rates by sex and education. Interestingly, there is less evidence of heterogeneity by race or ethnicity, despite the substantial differences in national overdose rate growth since 1996. These findings suggest that some of the racial and ethnic disparities at the national level occurred across both triplicate and non-triplicate states, reflecting the year fixed effects in the models discussed in Sect. 4.2.

To illustrate this interpretation, I present time series of overdose death rates (equivalent to Fig. 1) by triplicate status in Fig. 5. In triplicate states (Panel B), there is little change in the overdose death rate until the fentanyl crisis among the Black and Hispanic populations, but we see growth for these groups in non-triplicate states (Panel A) during these years. In contrast, the White and AI/AN populations experience increases in both triplicate and non-triplicate states; the growth is just faster in non-triplicate states. These figures highlight that some racial/ethnic overdose disparities emerged in both triplicate states and non-triplicate states.

For disparities by sex, there is little evidence of any growth in the gap between males and females in triplicate states after OxyContin’s introduction (Panel D) until the fentanyl crisis. For non-triplicate states (Panel C), there is evidence of large and growing disparities throughout the post-period.

Panels E and F provide overdose death rates by education. As with sex-specific overdose rates, there is little evidence of growing disparities due to OxyContin in triplicate states until the fentanyl crisis begins. In contrast, non-triplicate states experienced large and increasing education disparities throughout the 1996–2020 period.

Interestingly, for sex- and education-specific overdose death rates, there is little evidence of any change in disparities through the first two waves of the opioid crisis in triplicate states. Instead, these disparities emerge predominantly in non-triplicate states, underscoring the significant role OxyContin played in driving these disparities. There are also some notable disparities by race/ethnicity—the AAPI population specifically appears less impacted by the launch of OxyContin relative to all other racial/ethnic groups. However, we also observe evidence of disparities in triplicate states across some racial/ethnic groups, suggesting that other factors may be contributing to these differences. These factors could potentially include hidden (to us) practices related to the marketing of OxyContin, but further isolating these mechanisms is challenging.

## Conclusion and implications

The opioid crisis has engendered staggering demographic differentials in overdose rates. This paper provides evidence all demographic groups were impacted by the supply and promotion of OxyContin and experienced large overdose rate growth due to these factors. For many demographic factors, we generally observe larger overdose gaps between demographic groups in states more exposed to the opioid crisis due to an early and persistent supply shock. These overdose differentials persist even beyond the introduction of an abuse-deterrent version of OxyContin as states more exposed to OxyContin experienced sharper growth in their illicit opioid markets. Notably, this transition to illicit markets has also begun to alter the demographics of the opioid crisis in more recent years, especially in terms of the racial incidence (Friedman and Hansen 2022; Smith et al. 2024).

I implemented a simple decomposition exercise which uncovered that many disparities exist despite, not because of, other associated demographic characteristics. For example, the “whiteness” of the opioid crisis (Hansen et al. 2023) during the first wave can be observed by the relatively high rates of overdose deaths among White individuals relative to most other racial/ethnic groups (the AI/AN population is an important exception). However, the White population tends to be older and more likely to have a college education—both of which are typically protective factors against overdose death rate growth. This suggests that the high rate of overdose deaths among White individuals occurs *despite* these protective demographic correlates. Related, the relatively low rates of overdose deaths among Black and Hispanic individuals (during the first two waves) occur despite covarying characteristics which predict disproportionate exposure to the opioid crisis. I also find that low education groups have high overdose rates, but we would expect them to have even higher overdose death rates given their broader demographic profiles.

Thus, while many of the documented overdose disparities are vast, it is notable that they mask even larger adjusted differences. This finding highlights the potential scope for many hypothesized factors, such as discrimination in opioid prescribing during the first wave (Hansen et al. 2023; Swift et al. 2019), to have played elevated roles in driving overdose rate disparities given that the underlying disparities are even larger than the observed rates would suggest. These hypotheses deserve additional empirical scrutiny.

I also find that a major source of geographic variation in the opioid crisis plays a modest mechanical role in explaining national disparities. However, a complementary analysis finds a leading role for differential *responses* to the introduction of OxyContin for explaining some overdose death rate disparities. OxyContin appears to have induced large sex- and education-specific disparities as large differences are observed across demographic groups in terms of how they responded to its launch and promotion. In fact, prior to the fentanyl crisis, there is little evidence of any growth in sex- and education-specific disparities in states less exposed to the OxyContin’s launch. These disparities only emerged in non-triplicate states, highlighting the central role of OxyContin’s launch and promotion in driving these differences.

In contrast, there were rather uniform responses to OxyContin exposure for Black, Hispanic, and White Americans, despite very different national overdose trajectories for these groups. Studying overdose trends in triplicate states specifically, there is evidence of growing disparities when comparing White and AI/AN individuals to other racial and ethnic groups even in these less exposed states, suggesting the independent importance of other factors.

This paper finds that OxyContin’s introduction played a large role in creating and exacerbating disparities, most especially disparities by educational attainment. This finding leads to more questions about why some groups would be more impacted by this supply shock than others. The evidence that overdose differentials across demographic groups are supply-driven does not rule out the importance of demand-side factors. It is possible that different populations have varying demands for substance use. In such a case, a uniform opioid supply shock would differentially impact these populations, suggesting that the interaction of supply and demand factors is essential. Similarly, it is possible that within-state variation or non-geographic sources of variation (e.g., demographic differences in engagement with the healthcare system) in exposure to the introduction of OxyContin further explain the estimated disparities. The reasons behind this heterogeneity merit future research.

However, the results strongly suggest that OxyContin, and opioid access more generally, played an independent and critical role in the development and persistence of demographic-specific trends. The literature provides limited evidence to explain the reasons why some demographic groups have been more impacted by the opioid crisis than others. This paper finds that OxyContin’s launch—and its enduring impacts—played an essential role in the magnitudes of the overdose rate growth and the differences in that growth across many demographic groups. These findings represent an important first step in a literature with few empirical tests explaining the evolution of overdose disparities.

The results also suggest that the increase in opioid access through OxyContin’s launch and general promotion of opioids generated new overdose deaths across demographic groups, rather than simply crowding out other types of drug overdoses. Thus, while “despair” due to poor economic and cultural conditions may have played a synergistic role, this supply-side shock was a necessary factor in inducing the additional drug overdose deaths that have defined the opioid crisis. These supply-side impacts appear to have affected nearly all demographic groups.

Future work should consider why some demographic groups were more strongly affected by the supply shock than others. This paper provides evidence of differential geographic exposure to OxyContin since its introduction, but it is an open question why—for example—White Americans received higher rates of OxyContin prescribing than Black Americans, though the literature has suggested hypotheses (Om 2018; Netherland and Hansen 2017). However, given that there are such different responses to this major supply shock, this research suggests that studying the opioid crisis—especially the evaluation of supply-side interventions—merits separate analyses based on demographics. Estimating differential responses by race and ethnicity, sex, education, and age should be the norm when evaluating responses to policies targeting opioid use/misuse (see Townsend et al. 2022 for a nice example), though it is rarely done in practice. This paper begins thinking about how to test these hypotheses and to consider the foundational reasons for the development of the defining disparities generated by the opioid crisis.

Overall, few demographic groups escaped the supply-driven forces of the opioid crisis. This result is fundamental to understanding the underlying structural factors driving the opioid crisis across the population. Those structural factors may differ across groups, but the literature has generally not considered the role of supply-side shocks on overdose rate disparities. The launch of OxyContin and the accompanying aggressive promotion of strong opioids had extensive but disparate impacts across the population. These disparate impacts, in many cases, can explain existing differences in overdose rates across demographic groups. For example, the overdose rates of men relative to women grew faster in states more exposed to OxyContin’s launch; the overdose rates of those with no college education were substantially higher relative to those with some college education in those same states. This evidence implies that OxyContin’s launch and its enduring effects can partially explain the demographics of the overdose death rates in the United States.

## Supplementary Information

Below is the link to the electronic supplementary material.

Supplementary file 1 (pdf 510 KB)

## Data Availability

The mortality data used in the paper are restricted for the years 2005–2020. It is necessary to apply for data access. More information can be found here: https://www.cdc.gov/nchs/nvss/nvss-restricted-data.htm. The 1989–2004 files can be found here: https://www.cdc.gov/nchs/nvss/mortality_public_use_data.htm. The population data are here: https://seer.cancer.gov/popdata/. Education counts were created using the CPS IPUMS: https://cps.ipums.org/cps/.

## Footnotes

1 On race and ethnicity, also see Shiels et al. (2018); SAMHSA (2020); James and Jordan (2018); Singh et al. (2019); Stevens-Watkins (2020); Drake et al. (2020); Cano (2021); Whelshula et al. (2021); Friedman et al. (2021); Furr-Holden et al. (2021); Gondré-Lewis et al. (2022); Kariisa et al. (2022); Qeadan et al. (2022); Humphreys et al. (2022); Friedman et al. (2022); Friedman and Hansen (2022, 2024). Milam et al. (2021) study classification of opioid-involved overdose deaths over time and how improvements in classification have varied by race. On sex, also see Mack et al. (2013); McCarthy (2013), and Altekruse et al. (2020).

2 Case and Deaton (2015, 2017) argue that rising overdose death rates are a function of deteriorating economic and cultural conditions for large segments of the population. A growing literature studies how economic conditions or shocks to labor opportunities relate to overdoses or a broader set of deaths of despair (Hollingsworth et al. 2017; Ruhm 2019a; Venkataramani et al. 2020; Pierce and Schott 2020; Betz and Jones 2018; Charles et al. 2019; Currie et al. 2019; O’Brien et al. 2022). In addition, Ruhm (2019b) documents that non-opioid overdose death rates are also rapidly increasing, suggesting that the ongoing overdose epidemic is not specific to opioids.

3 For example, Jalal et al. (2018) study overdose death rates back to 1979, though the demographic analyses begin in 1999.

4 Purdue Pharma’s stated objective in the early years was as follows: “To convince health care professional (physicians, nurses, pharmacists, and managed health care professionals) to aggressively treat both non-cancer pain and cancer pain. The positive use of opioids, and OxyContin Tablets in particular, will be emphasized” (Purdue Pharma 1999).

5 This medical access may (and likely does) spill over into illicit markets (Powell et al. 2020; Schnell 2022).

6 Arteaga and Barone (2022) use cancer mortality rates as a proxy for MS Contin prescriptions. Cancer mortality does predict OxyContin adoption, though it is unclear whether this is due to independent demand for the drug (i.e., demand for pain management options among cancer patients) or Purdue Pharma’s marketing strategies.

7 Alpert et al. (2022) dedicate a significant amount of analysis to isolating the underlying mechanisms generating the large overall post-1996 overdose effects, determining that it is primarily driven by a marketing effect and not direct effects of triplicate programs impacting overdose rates even years after repeal of the programs. Relative to Alpert et al. (2022), there is little new evidence in this paper about the underlying mechanisms for *why* differential access to OxyContin after launch caused such long-term mortality effects.

8 The Open Payment Database collects and lists data on payments—for research, meals, travel, gifts, or speaking fees—from drug companies to physicians and teaching hospitals.

9 “McKinsey recommended doubling down on Purdue Pharma’s strategy of targeting high prescribers for even more sales calls...” (Commonwealth of Massachusetts 2018). Purdue Pharma ended promotional activities for OxyContin in 2018.

10 The online ARCOS data are provided by ingredient. I made a FOIA request for data on OxyContin specifically. This request was approved for 2000–2016.

11 I end the time series in 2005 since Medicare Part D was enacted in 2006. While the current SDUD available online suppresses numbers of prescriptions less than ten, I use a version downloaded before this suppression policy was implemented.

12 I accessed these data in the AHRQ Data Facility due to the necessity for geocoded data.

13 There is a notable dip in national OxyContin supply in 2005–2006 due to a patent dispute at this time. However, total oxycodone supply was unaffected over this period, presumably as generic OxyContin formulations substituted for the brand-name version (see Fig. A.3). This dispute was eventually resolved and the generic versions exited the market. Despite these large shifts in national trends, the relative supply of non-triplicates and triplicates remained approximately the same.

14 See the example provided in footnote 4.

15 Prior to 1989, some states reported ethnicity through different formats. Oklahoma, Louisiana, and New Hampshire did not report ethnicity until 1991 (Louisiana) to 1997 (Oklahoma). However, Hispanic origin is uncommon in these states among overdose deaths in the years after they begin reporting. Oklahoma is dropped from the analysis for reasons discussed below. I keep the other two states in the sample and simply assume that none of the overdose deaths prior to the state beginning to report ethnicity are among the Hispanic population. Excluding these states has little impact on the conclusions of this paper. In general, Hispanic origin reporting is “reasonably good” on death certificates (Arias et al. 2010).

16 See Table 2 of https://www.cdc.gov/drugoverdose/pdf/pdo_guide_to_icd-9-cm_and_icd-10_codes-a.pdf, last accessed August 18, 2021. I include opioid-involved overdose results in the Appendix. I use deaths involving E850.0, E850.1, E850.2, or N965.0 (Alexander et al. 2018) for these analyses.

17 The CDC reports that the transition from ICD-9 to ICD-10 resulted in a small increase in poisoning-related deaths (not necessarily drug poisonings) of 2% (Warner et al. 2011). When studying opioid-involved overdose deaths in the appendix, I use the T40.0-T40.4, T40.6 codes to limit overdoses to those involving opioids.

18 Ruhm (2018) discusses missing opioid designations on death certificates. Empirical work has often used the broader overdose category for these reasons (e.g., Venkataramani and Chatterjee 2019).

19 There are concerns about the accurate reporting of race for AI/AN individuals, leading to possible undercounts of overdose deaths (Joshi et al. 2018).

20 The individual categories change over time. This aggregated category creates a consistent group.

21 Noymer et al. (2011) find that cause of death can affect racial classification, which could bias some of the findings in this paper. Unfortunately, it is difficult to quantify overdose mortality over this full time period using alternative data sources.

22 I use these categories because I cannot determine high school graduation status prior to the 2003 revision. Case and Deaton (2017) divide the “college” category further. Alternatively, Leive and Ruhm (2022), Leive and Ruhm (2021), and Novosad et al. (2022) construct *relative* education groups based on within-year placement.

23 In cases in which overdose deaths are rare for that cell, I use the education distribution for the same state, year, race, ethnicity, and sex. For the remaining cases, I use the average of the education distribution in the previous and subsequent year (for a fixed state, race, ethnicity, sex, and age group).

24 These data only include ethnicity beginning in 1990 so I use Census ethnicity data for 1989.

25 Instead of constructing population levels directly in the CPS, I calculate the share of each cell (year $\times$ state $\times$ race/ethnicity $\times$ age group $\times$ sex) which did not attend college and multiply that fraction by the population size of the cell in the SEER data to estimate population sizes for each cell $\times$ education group.

26 See Tipps et al. (2018) and Whelshula et al. (2021) for important exceptions which report AI/AN overdose death rates back to 1999. In this paper, I extend these trends back to 1989.

27 Another possibility is to estimate a model in which only one set of racial/ethnic group $\times$ year indicators are included at a time. This approach also makes comparisons difficult since the baseline in such a case is all *other* racial/ethnic groups so the baseline is different for each group. For example, AI/AN individuals typically have higher overdose death rates than all other groups, but their rates would look smaller than the rate for White individuals since overdose death rates are high for White individuals, who compose the majority of the US population. White individuals would be part of the comparison for the AI/AN population, but they would not be included in the comparison group for themselves. The proposed approach fixes the value of the comparison across all groups.

28 In many economic settings, controlling for other variables often attenuates the differences in outcomes for vulnerable groups because the controls themselves are also impacted by social factors of interest. In this context, the controls are age, sex, and education where the latter is likely the only factor that could potentially reflect socioeconomic status which could be affected by discrimination and other social determinants. This exercise is descriptive, and the interest is understanding whether existing racial (or sex or education) disparities operate through differences in other factors or can be attributed solely to race.

29 In the absence of OxyContin, I cannot rule out that another addictive opioid might have been aggressively marketed at some point in time and generated large overdose effects. In this respect, this paper is a partial equilibrium analysis, using the launch of OxyContin as a proxy for exposure to the existing opioid crisis given the large differences in supply generated by its introduction.

30 I do not include additional covariates. Alpert et al. (2022) finds that results are similar for the overall overdose rates whether additional controls are included/excluded. Powell (2021) documents shifting demographics in the triplicate and non-triplicate states but shows that these systematic changes do not drive the mortality results.

31 There is a small pre-trend for the Hispanic population, but this trend flattened from 1995 to 1999. This population then experienced some growth in overdose rates in non-triplicate states relative to triplicate states but such a delayed impact would seem unlikely to be due to the pre-1995 trend.

32 For the averages between period $t_0$ and $t_1$, I use $\frac{1}{t_1 - t_0 + 1} \sum _{t_0}^{t_1} \hat{\beta }_t - \frac{1}{5} \sum _{t=1991}^{1995} \hat{\beta }_t$. As should be evident from the event study results, the choice of pre-period does not meaningfully impact the results. I use 1991–1995 instead of 1995 since, in principle, a longer pre-period will make the estimates less susceptible to transitory pre-period shocks. Alternatively, I could use the full pre-period (1989–1995) and results are similar.

33 Powell et al. (2023) discuss the role of reformulation in polysubstance deaths involving prescription non-opioid drugs.

34 Given the specification, this means that there are few untreated units, though the same problems arise as having few treated units. Traditional clustered (by state) standard errors using a cluster covariance matrix estimator typically produce much tighter confidence intervals in the context of this paper.

35 While overdose rates generally increased throughout this period among White and AI/AN individuals, growth was much less monotonic for the Black population, peaking in 2006 for the 1995–2009 period and subsequently declining for a few years.

36 These are aggregates of the unadjusted rates shown in Fig. 1 but presented relative to the mean and relative to the baseline period.

37 The one exception in Table 1 is that Black individuals experienced higher relative overdose rates in 2011–2020 compared to 2001–2010, reducing the magnitude of the disparity. As evident in Fig. A.5, the Black population began experiencing disproportionate overdose rate growth in 2016.

38 The higher share of White individuals in non-triplicate states explains about 4% of the higher overdose rate for White Americans.

39 For example, while 72% of the US population (for 1996–2020) lives in non-triplicate states, only 40% of the Hispanic population lives in non-triplicate states.
