# Supplementary material for: Understanding the demographics of the opioid overdose death crisis
Source: J Popul Econ. 2025 Jun 19;38(3):54. doi: 10.1007/s00148-025-01108-0 (PMC12179229; doi:10.1007/s00148-025-01108-0)
Supplement: Supplementary file 1 — (pdf 510 KB) [file 148_2025_1108_MOESM1_ESM.pdf]

A Appendix for "Understanding the demographics of the opioid overdose death crisis" by David Powell

Figure A.1: Example of Purdue Pharma Focus Group Recommendations

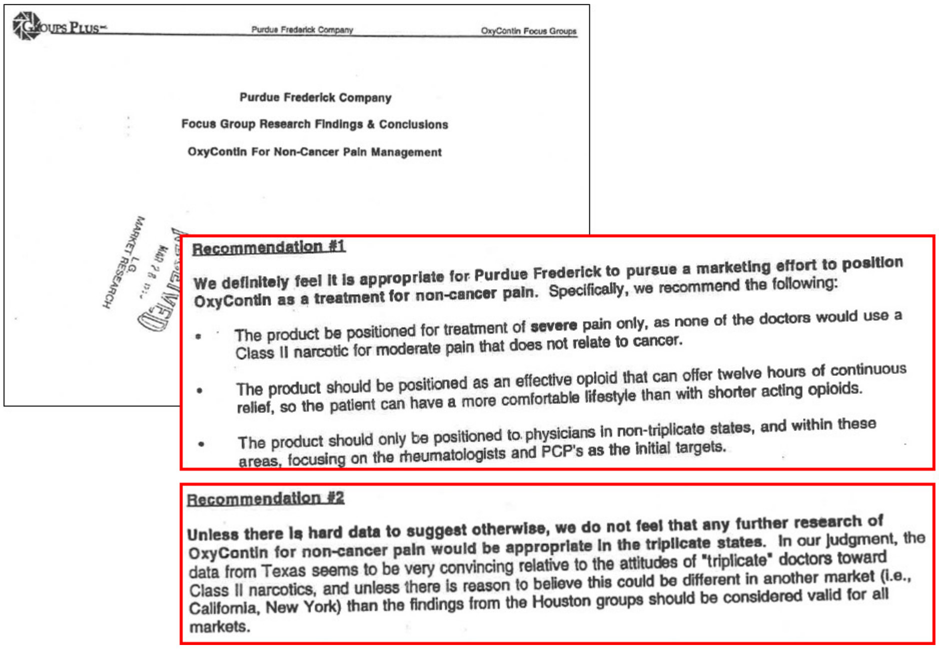

Groups Plus — Purdue Frederick Company — OxyContin Focus Groups

Purdue Frederick Company

Focus Group Research Findings & Conclusions

OxyContin For Non-Cancer Pain Management

Recommendation #1

We definitely feel it is appropriate for Purdue Frederick to pursue a marketing effort to position OxyContin as a treatment for non-cancer pain. Specifically, we recommend the following:

- The product be positioned for treatment of **severe** pain only, as none of the doctors would use a Class II narcotic for moderate pain that does not relate to cancer.
- The product should be positioned as an effective opioid that can offer twelve hours of continuous relief, so the patient can have a more comfortable lifestyle than with shorter acting opioids.
- The product should only be positioned to physicians in non-triplicate states, and within these areas, focusing on the rheumatologists and PCP's as the initial targets.

Recommendation #2

Unless there is hard data to suggest otherwise, we do not feel that any further research of OxyContin for non-cancer pain would be appropriate in the triplicate states. In our judgment, the data from Texas seems to be very convincing relative to the attitudes of "triplicate" doctors toward Class II narcotics, and unless there is reason to believe this could be different in another market (i.e., California, New York) than the findings from the Houston groups should be considered valid for all markets.

Notes: This figure shows a copy of and relevant passage from Groups Plus (1995).

Figure A.2: OxyContin Promotional Activity, Distribution, and Prescriptions by Triplicate State Status

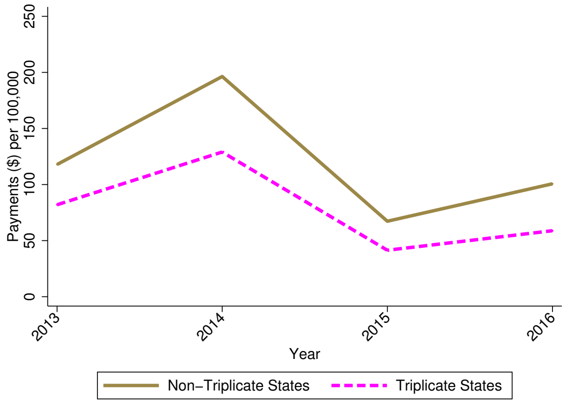

A: OxyContin Promotional Payments

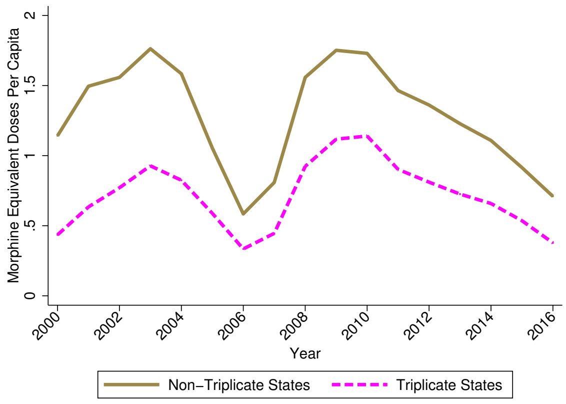

B: OxyContin Distribution (ARCOS)

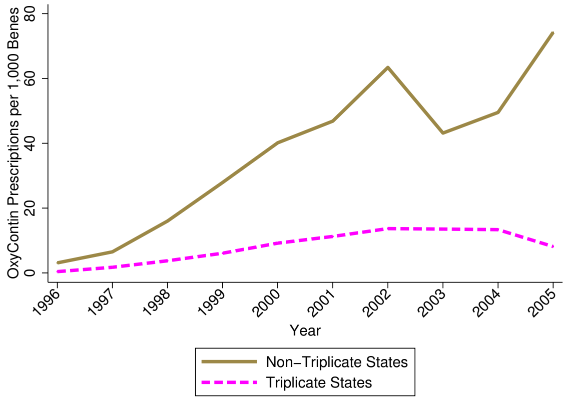

C: OxyContin Prescriptions (Medicaid)

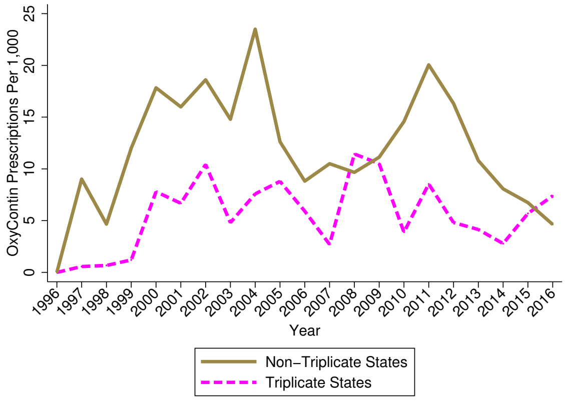

D: OxyContin Prescriptions (MEPS)

Notes: In Panel A, I use CMS Open Payments Data to calculate total payments and gifts made to physicians regarding OxyContin for the available years. I scaled this measure by population. The outcomes correspond to August 2013 – December 2016. Because the 2013 data only cover a partial year, I annualize the rate in that year. In Panel B, I use ARCOS data to construct OxyContin morphine equivalent doses per capita. I define a morphine equivalent dose as 60 morphine milligram equivalents. OxyContin data are only available for 2000-2016. In Panel C, I report the number of prescriptions per 1,000 beneficiaries from the Medicaid SDUD. I end this time series in 2005 due to the introduction of Medicare Part D. In Panel D, I report the number of prescriptions per 1,000 people in the MEPS using MEPS survey weights.

Figure A.3: Oxycodone Distribution Triplicate State Status

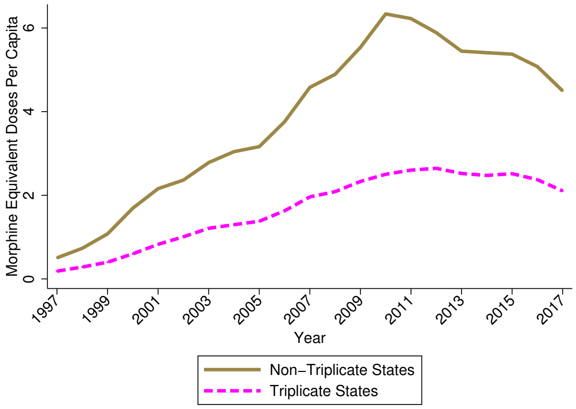

Oxycode Distribution (ARCOS)

Notes: I use ARCOS data to construct oxycodone morphine equivalent doses per capita. I define a morphine equivalent dose as 60 morphine milligram equivalents. Oxycodone data are only available starting in 1997.

Figure A.4: Drug Overdose Death Rates for 1989-2020 by Demographic Group (for analysis sample)

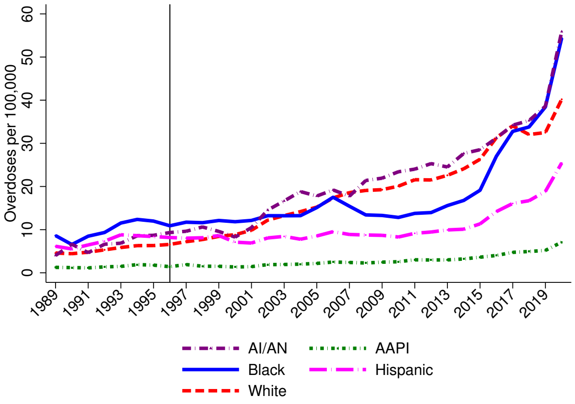

A: Race and Ethnicity

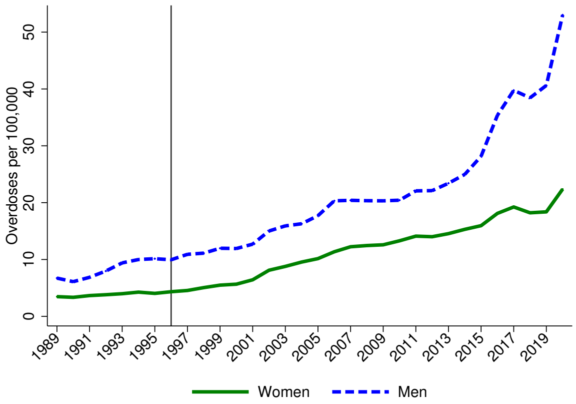

B: Sex

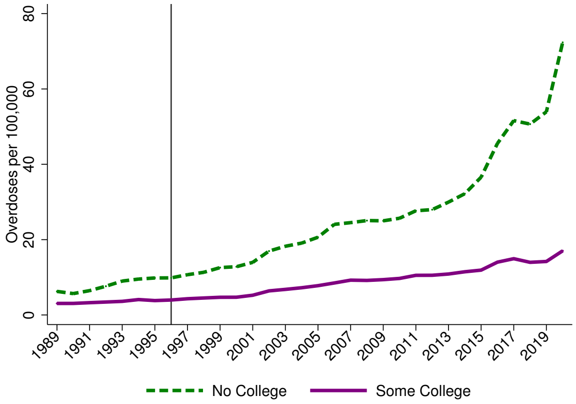

C: Education

Notes: I plot drug overdose death rates for 1989-2020 using NVSS data. Georgia, Oklahoma, Rhode Island, and South Dakota are excluded because of missing education information early in the sample period. Panel C is identical to the one in Figure 1. For 1989-1998, I define drug poisonings as deaths involving underlying cause of death ICD-9 codes E850-E858, E950.0-E950.5, E962.0, or E980.0-E980.5. For the 1999-2018 data, I code deaths as drug overdoses using the ICD-10 external cause of injury codes X40-X44, X60-64, X85, or Y10-Y14. Ethnicity and education information are only available beginning in 1989. "Some college" means at least some college education. Overdose deaths are scaled by population using Census, SEER, and CPS data (see text for details).

Figure A.5: Decomposition Exercise by Race and Ethnicity

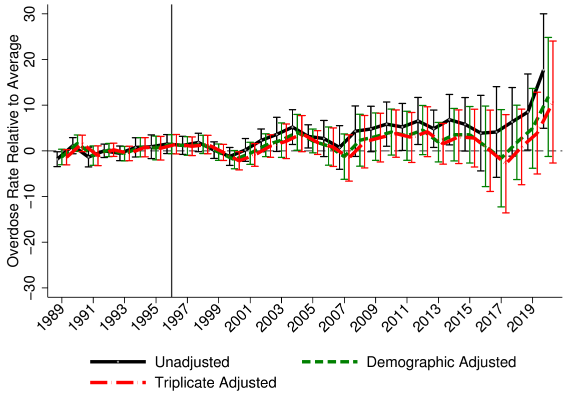

A: AI/AN

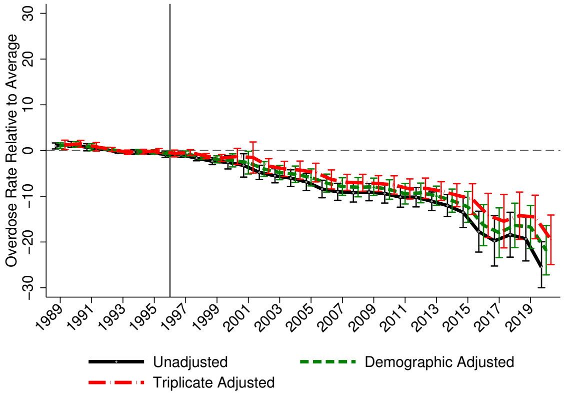

B: AAPI

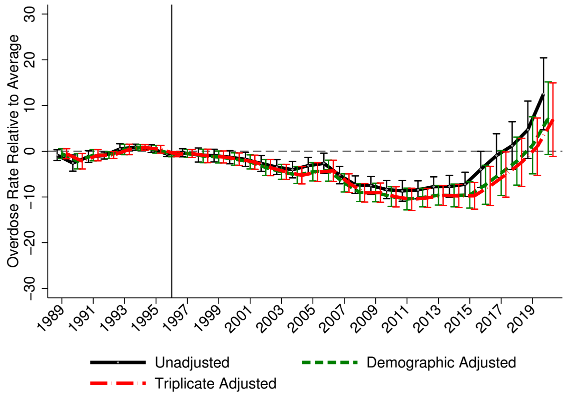

C: Black

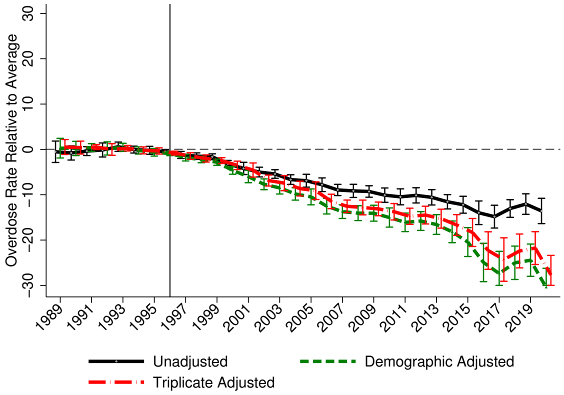

D: Hispanic

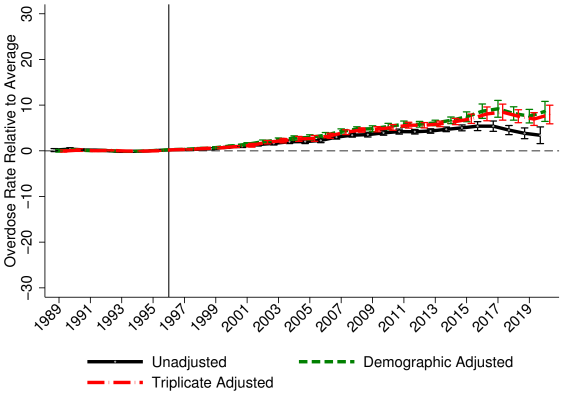

E: White

Notes: Panels A-E provide decomposition estimates from estimation of equation (1) in the paper. The estimates represent the overdose death rate for that group *relative to the average* in that year. Unlike the results shown in Table 1, these estimates are *not* relative to 1991-1995. 95% confidence intervals are adjusted for within-state clustering. Confidence intervals are truncated at -30 and 30 to improve readability of the figures.

Figure A.6: Decomposition Exercise by Sex and Education

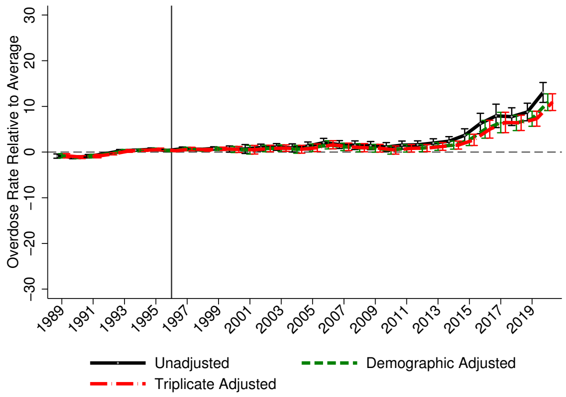

A: Men

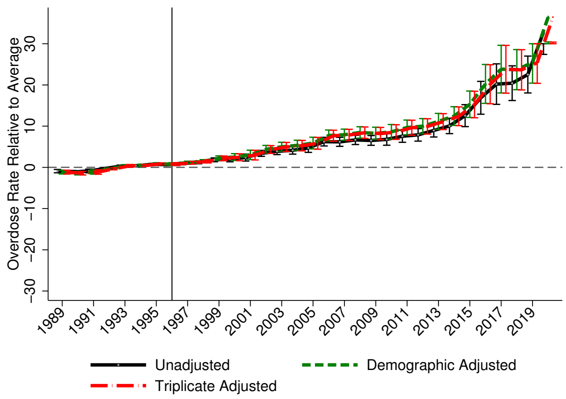

B: No College

Notes: Panels A-B provide decomposition estimates from estimation of equation (1) in the paper. The estimates represent the overdose death rate for that group *relative to the average* in that year. Unlike the results shown in Table 2, these estimates are *not* relative to 1991-1995. 95% confidence intervals are adjusted for within-state clustering. Confidence intervals are truncated at -30 and 30 in Panel B to improve readability of the figures.

Tables

Table A.1: Descriptive Statistics by Race and Ethnicity

Panel A: No College

Period	AI/AN (%)	AAPI (%)	Black (%)	Hispanic (%)	White (%)
1991-1995	62.96	41.21	64.55	73.01	52.37
1996-2000	57.02	37.42	58.24	70.96	47.12
2001-2010	52.63	32.42	53.45	68.20	41.91
2011-2020	48.28	28.71	45.97	61.07	35.17
**1996-2020**	**51.41**	**31.17**	**50.90**	**64.99**	**40.13**

Panel B: Male

Period	AI/AN (%)	AAPI (%)	Black (%)	Hispanic (%)	White (%)
1991-1995	47.89	47.16	44.97	50.16	47.63
1996-2000	48.06	47.02	45.26	50.46	47.83
2001-2010	48.04	46.65	45.59	50.18	48.15
2011-2020	48.31	46.78	46.44	50.26	48.81
**1996-2020**	**48.16**	**46.76**	**45.92**	**50.26**	**48.36**

Panel C: Ages 25-44

Period	AI/AN (%)	AAPI (%)	Black (%)	Hispanic (%)	White (%)
1991-1995	60.14	60.14	56.96	65.03	47.29
1996-2000	56.82	56.81	54.51	64.24	44.20
2001-2010	48.70	52.38	48.25	60.25	38.06
2011-2020	43.44	48.04	44.23	53.69	34.11
**1996-2020**	**47.69**	**50.74**	**47.51**	**57.49**	**37.61**

Panel D: Ages 45-64

Period	AI/AN (%)	AAPI (%)	Black (%)	Hispanic (%)	White (%)
1991-1995	29.69	28.73	28.17	25.06	30.80
1996-2000	33.02	31.42	31.02	26.10	33.87
2001-2010	39.44	34.66	37.28	29.82	39.74
2011-2020	39.77	35.50	38.90	34.24	39.89
**1996-2020**	**38.52**	**34.67**	**36.94**	**31.55**	**38.68**

Panel E: Ages 65+

Period	AI/AN (%)	AAPI (%)	Black (%)	Hispanic (%)	White (%)
1991-1995	10.17	11.13	14.87	9.90	21.91
1996-2000	10.16	11.76	14.47	9.65	21.93
2001-2010	11.86	12.97	14.47	9.93	22.20
2011-2020	16.80	16.46	16.87	12.07	26.00
**1996-2020**	**13.79**	**14.59**	**15.55**	**10.97**	**23.71**

Notes: This table provides descriptive statistics for the aged 25+ population by race/ethnicity for educational attainment, sex, and age. For example, the first column of Panel A provides the percentage of the AI/AN population (aged 25+) without any college experience.

Table A.2: Descriptive Statistics by Sex

Panel A: Female

**Period**	**AI/AN (%)**	**AAPI (%)**	**Black (%)**	**Hispanic (%)**	**White (%)**	**Ages 25-44 (%)**	**Ages 45-64 (%)**	**Ages 65+ (%)**	**No College (%)**
1991-1995	0.54	3.16	10.76	7.84	77.70	48.00	29.55	22.46	57.36
1996-2000	0.60	3.87	11.10	9.20	75.23	45.78	32.22	22.00	51.88
2001-2010	0.64	5.01	11.43	11.82	71.09	41.06	37.48	21.46	46.14
2011-2020	0.67	6.41	12.00	14.61	66.31	37.75	38.17	24.08	38.81
**1996-2020**	**0.64**	**5.41**	**11.62**	**12.55**	**69.78**	**40.48**	**36.83**	**22.69**	**44.02**

Panel B: Male

**Period**	**AI/AN (%)**	**AAPI (%)**	**Black (%)**	**Hispanic (%)**	**White (%)**	**Ages 25-44 (%)**	**Ages 45-64 (%)**	**Ages 65+ (%)**	**No College (%)**
1991-1995	0.55	3.11	9.70	8.70	77.94	52.67	30.47	16.86	52.46
1996-2000	0.60	3.76	10.03	10.24	75.37	50.00	33.27	16.74	48.57
2001-2010	0.64	4.74	10.36	12.88	71.38	44.42	38.56	17.02	45.88
2011-2020	0.66	5.95	11.00	15.60	66.79	40.63	39.16	20.20	41.27
**1996-2020**	**0.64**	**5.09**	**10.58**	**13.60**	**70.08**	**43.75**	**37.89**	**18.36**	**44.34**

Notes: This table provides descriptive statistics for the female and male populations. For example, the first column in Panel A represents the percentage of the female population identifying as AI/AN during each period.

Table A.3: Descriptive Statistics by Education

Panel A: No College

Period	AI/AN (%)	AAPI (%)	Black (%)	Hispanic (%)	White (%)	Ages 25-44 (%)	Ages 45-64 (%)	Ages 65+ (%)	Male (%)
1991-1995	0.62	2.35	12.03	10.94	74.05	43.45	30.51	26.04	45.33
1996-2000	0.68	2.84	12.26	13.68	70.54	42.46	31.48	26.06	46.14
2001-2010	0.73	3.44	12.68	18.27	64.88	38.53	35.89	25.58	47.90
2011-2020	0.80	4.44	13.23	23.04	58.50	34.61	38.76	26.63	50.16
**1996-2020**	**0.75**	**3.71**	**12.81**	**19.21**	**63.52**	**37.79**	**36.12**	**26.09**	**48.43**

Panel B: Some College

Period	AI/AN (%)	AAPI (%)	Black (%)	Hispanic (%)	White (%)	Ages 25-44 (%)	Ages 45-64 (%)	Ages 65+ (%)	Male (%)
1991-1995	0.45	4.10	8.08	4.95	82.41	58.50	29.34	12.16	50.28
1996-2000	0.52	4.81	8.89	5.66	80.11	53.19	33.98	12.83	49.45
2001-2010	0.56	6.11	9.42	7.26	76.65	46.21	39.80	14.00	48.17
2011-2020	0.57	7.35	10.37	9.80	71.91	42.18	38.58	19.24	47.60
**1996-2020**	**0.56**	**6.48**	**9.78**	**8.19**	**74.99**	**45.44**	**38.31**	**16.25**	**48.11**

Notes: This table presents descriptive statistics for populations categorized by educational attainment (No College and Some College). For example, the first column in Panel A indicates the percentage of the population with no college education identifying as AI/AN in each period.

Table A.4: Differences in Demographics by Triplicate Status

Panel A: No College

Period	Triplicate (%)	Non-Triplicate (%)
1991-1995	52.17	56.37
1996-2000	48.29	51.23
2001-2010	44.81	46.59
2011-2020	39.77	40.12
**1996-2020**	**43.22**	**44.63**

Panel B: Male

Period	Triplicate (%)	Non-Triplicate (%)
1991-1995	47.93	47.38
1996-2000	48.08	47.64
2001-2010	48.23	47.96
2011-2020	48.80	48.54
**1996-2020**	**48.45**	**48.15**

Panel C: AI/AN

Period	Triplicate (%)	Non-Triplicate (%)
1991-1995	0.41	0.61
1996-2000	0.44	0.67
2001-2010	0.44	0.73
2011-2020	0.43	0.78
**1996-2020**	**0.43**	**0.74**

Panel D: AAPI

Period	Triplicate (%)	Non-Triplicate (%)
1991-1995	5.73	1.93
1996-2000	6.84	2.41
2001-2010	8.41	3.22
2011-2020	10.23	4.24
**1996-2020**	**8.93**	**3.52**

Panel E: Black

Period	Triplicate (%)	Non-Triplicate (%)
1991-1995	9.90	10.42
1996-2000	10.08	10.82
2001-2010	10.02	11.34
2011-2020	10.23	12.13
**1996-2020**	**10.12**	**11.59**

Panel F: Hispanic

Period	Triplicate (%)	Non-Triplicate (%)
1991-1995	17.16	4.07
1996-2000	19.57	5.10
2001-2010	23.32	7.16
2011-2020	26.93	9.42
**1996-2020**	**24.24**	**7.76**

Panel G: White

Period	Triplicate (%)	Non-Triplicate (%)
1991-1995	66.80	82.98
1996-2000	63.07	80.99
2001-2010	57.81	77.55
2011-2020	52.18	73.44
**1996-2020**	**56.27**	**76.39**

Notes: This table provides descriptive statistics by 1996 triplicate status for educational attainment, sex, and race/ethnicity. These statistics refer to the aged 25+ population.

Table A.5: Decomposition Results by Race and Ethnicity: Isolating Importance of Covariates

Panel A: AI/AN

**Period**	**Unadjusted**	**Age Constant**	**Sex Constant**	**Educ Constant**
1996-2000	0.662	0.400	0.671	0.542
	[-0.481, 1.806]	[-0.755, 1.555]	[-0.475, 1.817]	[-0.610, 1.694]
2001-2010	3.346**	2.533*	3.373**	2.692*
	[0.471, 6.221]	[-0.442, 5.508]	[0.497, 6.249]	[-0.234, 5.617]
2011-2020	7.153**	6.048*	7.186**	4.622
	[0.671, 13.635]	[-0.627, 12.723]	[0.704, 13.667]	[-2.058, 11.301]
**1996-2020**	4.831**	4.112**	4.805**	3.294
	[0.929, 8.734]	[0.093, 8.131]	[0.896, 8.714]	[-0.723, 7.312]

Panel B: AAPI

**Period**	**Unadjusted**	**Age Constant**	**Sex Constant**	**Educ Constant**
1996-2000	-1.684***	-1.764***	-1.635***	-1.391***
	[-2.303, -1.065]	[-2.476, -1.053]	[-2.257, -1.013]	[-1.934, -0.847]
2001-2010	-7.203***	-7.735***	-7.107***	-5.791***
	[-9.021, -5.386]	[-9.835, -5.634]	[-8.919, -5.295]	[-7.360, -4.222]
2011-2020	-15.847***	-17.416***	-15.769***	-12.830***
	[-19.538, -12.157]	[-21.726, -13.105]	[-19.449, -12.090]	[-15.968, -9.692]
**1996-2020**	-9.776***	-10.099***	-9.976***	-8.339***
	[-12.219, -7.332]	[-12.806, -7.392]	[-12.460, -7.491]	[-10.557, -6.121]

Panel C: Black

**Period**	**Unadjusted**	**Age Constant**	**Sex Constant**	**Educ Constant**
1996-2000	-0.803	-0.956*	-0.776	-0.907*
	[-1.865, 0.258]	[-1.994, 0.081]	[-1.845, 0.294]	[-1.922, 0.109]
2001-2010	-4.679***	-5.355***	-4.639***	-5.359***
	[-6.361, -2.996]	[-7.084, -3.627]	[-6.316, -2.962]	[-7.057, -3.660]
2011-2020	-2.212	-3.702*	-2.098	-3.840**
	[-5.909, 1.484]	[-7.469, 0.064]	[-5.793, 1.598]	[-7.527, -0.153]
**1996-2020**	-2.694**	-3.512***	-2.679**	-3.828***
	[-5.077, -0.310]	[-5.932, -1.093]	[-5.062, -0.296]	[-6.205, -1.452]

Panel D: Hispanic

**Period**	**Unadjusted**	**Age Constant**	**Sex Constant**	**Educ Constant**
1996-2000	-1.638***	-1.856***	-1.651***	-2.335***
	[-2.199, -1.078]	[-2.368, -1.344]	[-2.199, -1.104]	[-2.883, -1.788]
2001-2010	-7.379***	-8.560***	-7.374***	-10.150***
	[-8.544, -6.215]	[-9.692, -7.428]	[-8.526, -6.221]	[-11.392, -8.908]
2011-2020	-12.057***	-15.305***	-12.272***	-18.723***
	[-13.846, -10.268]	[-17.295, -13.315]	[-14.058, -10.485]	[-21.041, -16.405]
**1996-2020**	-7.954***	-9.439***	-8.311***	-12.987***
	[-9.213, -6.696]	[-10.661, -8.217]	[-9.558, -7.064]	[-14.516, -11.458]

Panel E: White

**Period**	**Unadjusted**	**Age Constant**	**Sex Constant**	**Educ Constant**
1996-2000	0.375***	0.459***	0.373***	0.489***
	[0.216, 0.534]	[0.308, 0.609]	[0.215, 0.531]	[0.338, 0.641]
2001-2010	2.382***	2.812***	2.376***	2.944***
	[1.992, 2.772]	[2.376, 3.248]	[1.986, 2.766]	[2.522, 3.366]
2011-2020	4.380***	5.675***	4.415***	6.043***
	[3.592, 5.168]	[4.723, 6.626]	[3.624, 5.206]	[5.157, 6.928]
**1996-2020**	2.512***	3.045***	2.600***	3.621***
	[2.038, 2.985]	[2.525, 3.566]	[2.124, 3.076]	[3.107, 4.135]

Notes: ***Significance 1%, **Significance 5%, *Significance 10%. Outcome is all drug overdose deaths per 100,000 for the listed demographic. I report predictions relative to 1991-1995 and relative to the mean change in overdose deaths over all demographic groups. Column 1 shows unadjusted rates. Column 2 holds the age composition constant. Column 3 holds the share male constant. Column 4 holds educational attainment constant. Adjustments are not cumulative – when education is held constant, only education is held constant while age and sex are allowed to vary. 95% confidence intervals reported in brackets are adjusted for state-level dependence.

Table A.6: Decomposition Results by Sex and Educational Attainment: Isolating Importance of Covariates

**Panel A: Male**					**Panel B: No College**				
**Period**	**Unadjusted**	**Age Constant**	**Race Constant**	**Educ Constant**	**Period**	**Unadjusted**	**Age Constant**	**Sex Constant**	**Race Constant**
1996-2000	0.623***	0.548***	0.627***	0.617***	1996-2000	1.322***	1.496***	1.292***	1.457***
	[0.287, 0.959]	[0.235, 0.861]	[0.290, 0.965]	[0.262, 0.972]		[1.006, 1.638]	[1.125, 1.868]	[0.969, 1.615]	[1.123, 1.792]
2001-2010	1.284***	1.020***	1.282***	1.130**	2001-2010	5.031***	5.602***	4.910***	5.986***
	[0.438, 2.130]	[0.250, 1.791]	[0.438, 2.126]	[0.246, 2.015]		[4.081, 5.981]	[4.458, 6.745]	[3.933, 5.887]	[4.984, 6.987]
2011-2020	5.637***	5.101***	5.646***	4.969***	2011-2020	15.548***	16.499***	15.334***	17.654***
	[4.116, 7.159]	[3.731, 6.471]	[4.126, 7.166]	[3.497, 6.441]		[12.523, 18.573]	[13.116, 19.882]	[12.306, 18.362]	[14.487, 20.822]
**1996-2020**	3.117***	2.797***	3.096***	2.737***	**1996-2020**	7.817***	8.097***	7.828***	9.367***
	[2.125, 4.109]	[1.892, 3.701]	[2.107, 4.085]	[1.754, 3.721]		[6.344, 9.290]	[6.494, 9.700]	[6.311, 9.345]	[7.836, 10.898]

Notes: ***Significance 1%, **Significance 5%, *Significance 10%. Outcome is all drug overdose deaths per 100,000 for the listed demographic. I report predictions relative to 1991-1995 and relative to the mean change in overdose deaths over all demographic groups. Column 1 shows unadjusted rates. Column 2 holds the age composition constant. Column 3 holds race/ethnicity (Panel A) or the share male (Panel B) constant. Column 4 holds educational attainment (Panel A) or race/ethnicity (Panel B) constant. Adjustments are not cumulative – when education is held constant, only education is held constant while age and sex are allowed to vary. 95% confidence intervals reported in brackets are adjusted for state-level dependence.

Table A.7: Difference-in-Differences Estimates by Demographic Group: Opioid-Related Overdose Deaths

	Race and Ethnicity					Sex		Education	
**Period**	**AI/AN**	**AAPI**	**Black**	**Hispanic**	**White**	**Men**	**Women**	**No College**	**Some College**
1996-2000	2.268*	0.11	1.624	1.654***	0.336	1.349**	0.184	0.894	0.279*
	[-0.678, 5.409]	[-0.097, 0.262]	[-1.834, 4.915]	[0.554, 3.054]	[-0.362, 1.218]	[0.068, 2.970]	[-0.140, 0.711]	[-0.513, 2.903]	[-0.025, 0.542]
2001-2010	4.450**	0.502**	4.201	3.053*	2.962**	5.769***	2.290***	6.388***	1.391***
	[0.063, 8.849]	[0.050, 1.018]	[-0.473, 9.472]	[-0.393, 5.555]	[0.831, 5.062]	[1.872, 8.457]	[1.050, 3.650]	[2.297, 9.444]	[0.517, 2.085]
2011-2020	7.398	0.973***	8.845	7.624***	8.384***	13.905***	6.526***	19.259***	3.539***
	[-3.313, 18.030]	[0.618, 1.278]	[-7.461, 16.976]	[3.497, 12.001]	[2.547, 13.158]	[3.169, 21.168]	[3.123, 8.948]	[4.676, 28.671]	[0.462, 5.401]
**1996-2020**	5.193*	0.612***	5.543	4.602***	4.606***	8.139***	3.563***	10.437***	2.028***
	[-0.228, 10.815]	[0.417, 0.747]	[-2.959, 10.604]	[1.915, 7.280]	[2.343, 6.619]	[2.844, 11.770]	[2.080, 4.600]	[2.906, 14.742]	[0.768, 2.927]

Notes: ***Significance 1%, **Significance 5%, *Significance 10%. Outcome is drug overdose deaths involving opioids per 100,000 for the listed demographic. I estimate an event study, conditioning on state and year fixed effects. The event study estimates refer to the additional overdoses experienced in non-triplicate states by year. I average these estimates for the years listed in the first column. Estimates are relative to the pre-period, 1991-1995. 95% confidence intervals reported in brackets are estimated by clustered (by state) wild bootstrap.
